# Electronic and Vibrational Manifold of Tetracyanoethylene–Chloronaphthalene Charge Transfer Complex in Solution: Insights from TD-DFT and Ab Initio Molecular Dynamics

**DOI:** 10.1021/acs.jpca.2c05001

**Published:** 2022-09-29

**Authors:** Federico Coppola, Paola Cimino, Fulvio Perrella, Luigi Crisci, Alessio Petrone, Nadia Rega

**Affiliations:** † Department of Chemical Sciences, University of Napoli Federico II, Complesso Universitario di M.S. Angelo, 80126 Napoli, Italy; ‡ Scuola Superiore Meridionale, Largo San Marcellino 10, 80138 Napoli, Italy; § Department of Pharmaceutical Sciences, University of Salerno, 84084 Fisciano, Italy; ∥ Istituto Nazionale di Fisica Nucleare, Sezione di Napoli, Complesso Universitario di Monte S. Angelo ed. 6, 80126 Napoli, Italy; ⊥ Centro Interdipartimentale di Ricerca sui Biomateriali (CRIB), Piazzale Tecchio, 80125 Napoli, Italy

## Abstract

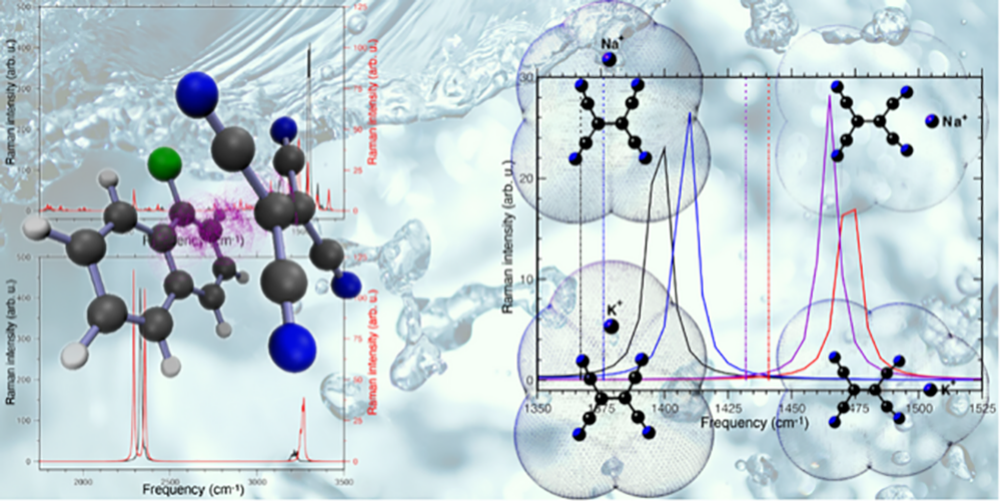

The interplay between light absorption and the molecular environment has a central role in the observed photophysics of a wide range of photoinduced chemical and biological phenomena. The understanding of the interplay between vibrational and electronic transitions is the focus of this work, since it can provide a rationale to tune the optical properties of charge transfer (CT) materials used for technological applications. A clear description of these processes poses a nontrivial challenge from both the theoretical and experimental points of view, where the main issue is how to accurately describe and probe drastic changes in the electronic structure and the ultrafast molecular relaxation and dynamics. In this work we focused on the intermolecular CT reaction that occurs upon photon absorption in a π-stacked model system in dichloromethane solution, in which the 1-chloronaphthalene (1ClN) acts as the electron donor and tetracyanoethylene (TCNE) is the electron acceptor. Density functional theory calculations have been carried out to characterize both the ground-state properties and more importantly the low-lying CT electronic transition, and excellent agreement with recently available experimental results [MathiesR. A.; et al. J. Phys. Chem. A2018, 122 ( (14), ), 35942955880210.1021/acs.jpca.8b00318] was obtained. The minima of the ground state and first singlet excited state have been accurately characterized in terms of spatial arrangements and vibrational Raman frequencies, and the CT natures of the first two low-lying electronic transitions in the absorption spectra have been addressed and clarified too. Finally, by modeling the possible coordination sites of the TCNE electron acceptor with respect to monovalent ions (Na^+^, K^+^) in an implicit solution of acetonitrile, we find that TCNE can accommodate a counterion in two different arrangements, parallel and orthogonal to the C=C axis, leading to the formation of a contact ion pair. The nature of the counterion and its relative position entail structural modifications of the TCNE radical anion, mainly the central C=C and C≡N bonds, compared to the isolated case. An important red shift of the C=C stretching frequency was observed when the counterion is orthogonal to the double bond, to a greater extent for Na^+^. On the contrary, in the second case, where the counterion ion lies along the internuclear C=C axis, we find that K^+^ polarizes the electron density of the double bond more, resulting in a greater red shift than with Na^+^.

## Introduction

**Figure 1:**
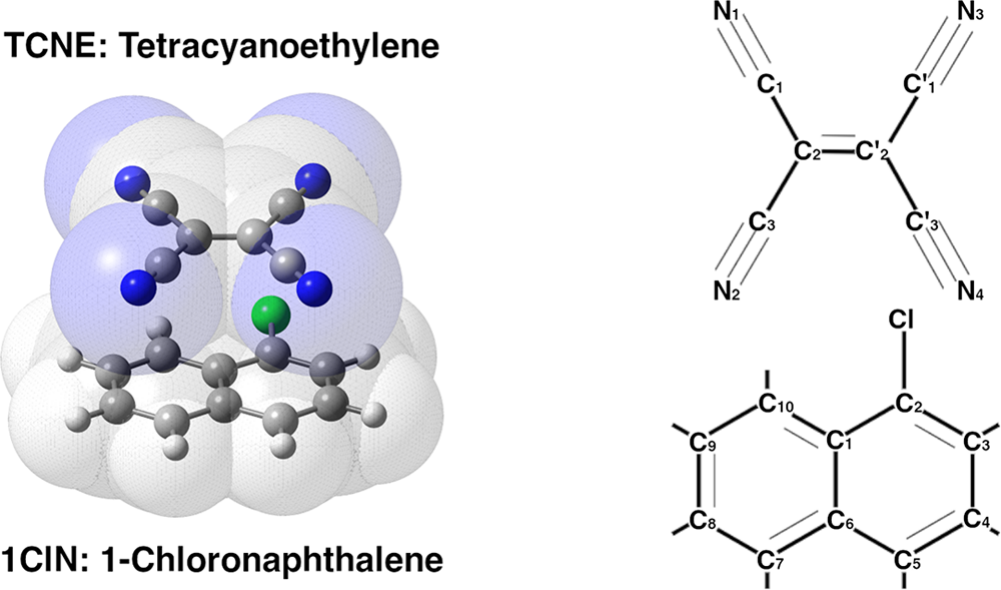
TCNE:π:1ClN charge transfer dimer in implicit DCM solvent (left) and schematic structure and labeling scheme (right).

Naturally occurring photoinduced phenomena that take place on the ultrafast (subpicosecond) time scale are as fascinating as they are extremely challenging to untangle and control. Achieving a deep knowledge of the key processes underlying photoreactive events is a core issue in both the spectroscopic and theoretical chemistry fields. Photoinduced charge transfer (CT) is a ubiquitous process of paramount importance in photochemistry,^1,2^ biology,^3,4^ and molecular electronic devices.^5−10^ Over the last years the scientific community has devoted a lot of effort to the rational design and fabrication of new compounds for light-harvesting applications to supplant the use of fossil fuels and rare-earth metals by favoring more sustainable materials. Systems ruled by charge separation are routinely employed because of their high versatility in many fields, such as organic optoelectronics,^11^ solar energy conversion,^12−15^ and nonlinear optics.^16−18^ Photoinduced CT can take place within the same molecular core,^19−22^ between space-separated species,^23,24^ at interfaces,^25,26^ in conjugated polymers,^10,27^ and in metal–ligand coordination complexes.^28−31^ Creating high-performance, stable, and efficient devices requires a detailed understanding of the molecular basis of the CT dynamics in materials. To have a broad and in-depth knowledge of the subtle electronic and nuclear interplay that strongly affects their efficiency, a joint approach between highly accurate computational methods and time-resolved spectroscopy techniques is mandatory. Photoinduced CT phenomena usually involve a complex interplay between electronic and vibrational coordinates, and to better disentangle these multiple effects and study of out-of-equilibrium dynamics, model systems are required for both methodological validations and development purposes. In this work, we focus on a non-covalent CT complex in which 1-chloronaphthalene (1ClN) acts as the electron donor (D) transferring electron density toward tetracyanoethylene (TCNE) as the electron acceptor (A) upon photoexcitation. The TCNE:π:1ClN complex embedded in the solvent cavity is represented in Figure 1.

The instant reorganization following photoexcitation of the electron cloud entails the opening of multiple competitive relaxation pathways over time, where both the vibrational and electronic manifolds play a major role. What are the determining factors that make one path preferential over another and how is it possible to break an ultrafast and unwanted decay are nontrivial questions that must be thoroughly addressed. The pioneering experimental investigation of this system by Mathies and co-workers using femtosecond stimulated Raman spectroscopy^23^ shed light on the relaxation mechanism of the TCNE:π:1ClN dimer, hypothesizing that the relaxation dynamics toward the electronic ground state is dictated by specific vibrational modes of the photoexcited electronic state. This vibrational dynamics represents the fingerprints of the charge recombination event. An accurate theoretical counterpart in agreement with the experimental findings was recently proposed by some of the present authors,^32^ in which the photorelaxation on the first singlet excited state was disentangled at the molecular level by the use of state-of-the-art methodologies based on Born–Oppenheimer molecular dynamics simulations to sample the excited-state potential energy surface (PES) and the multiresolution wavelet protocol to perform time-resolved vibrational analysis. From a methodological point of view, the theoretical approach required for a reliable description of such systems must simultaneously deal with accurate modeling of the weak interaction forces and the CT nature of all involved electronic states along with a careful description of the solvent effects. Currently a large number of electronic structure methods have been constantly revised and refined for computing the properties of ground and excited electronic states. Density functional theory (DFT), which encompasses a series of methods based on the knowledge of electron density,^33^ and its time-dependent version (TDDFT),^34−36^ for the calculation of excited-state properties, have proved to be valid methods for a wide variety of chemical problems due to the competitive accuracy of the results and the lower computational cost.^37−44^ The class of systems affordable via DFT can now range from isolated small molecules up to biological macromolecules and systems of technological relevance in materials science. We found that the central C=C stretching mode localized on the TCNE unit is particularly sensitive to the sudden electron density rearrangement, undergoing a relevant red shift in frequency (from 1530 to 1483 cm^–1^). Also, it plays a fundamental role in modulating the energy gap between the ground and CT electronic states. The out-of-plane bending mode, below 200 cm^–1^, it is mainly responsible for the separation of the two molecular planes, a further determining factor that modulates nonradiative decay processes. The characterization of the ground-state surface represented the first step to be faced for a reliable description of accessible conformational basins from which starting points for a swarm of excited-state ab initio molecular dynamics (AIMD) trajectories can be carefully selected and for the simulation of the absorption spectrum in solution accounting for the electrostatic interactions with the bulk solvent and thermal broadening.

As a corollary, in the present study we accurately describe both the electronic and vibrational manifolds, evaluating the major structural changes of the complexes following the transition between electronic states and how the electron density rearrangement affects the main vibrational features. The effect of thermal equilibrium at finite temperature on the spectroscopic absorption is highlighted via the computation of the electronic transitions on several snapshots extracted from AIMD, which were found to be in good agreement with the room-temperature UV–vis spectrum. A comparison of the minimum-energy geometries with respect to the distributions of ad hoc structural parameters calculated by the AIMD trajectory in the ground state is also given. Further insights on the vibrational manifold of the system are also provided. As a matter of fact, the possible coordination sites of TCNE in the presence of two cations evaluated under equilibrium conditions both from a structural and vibrational point of view allowed us to elucidate for the first time the origin of the splitting of the C=C stretching band that was experimentally measured for the tetracyanoethylene anion radical.^23^

In summary, as main results we find that the ground-state PES is quite flat and that numerous isoenergetic orientational isomers are present in solution. This justifies the employment of several snapshots from AIMD simulations to better describe the optical behavior of the system at finite temperature. In addition, the sudden electronic rearrangement upon excitation induces structural changes that affect both monomers and increase the charge separation, as quantified through population analysis and specific indices, reducing the intermolecular distance between the two subunits. A metal cation in the solvation sphere of TCNE can be hosted in the molecular plane along the C=C internuclear axis or orthogonal to it. The two arrangements are not equivalent, and different polarizations of the double bond result, as shown by the splitting of the related bands in the Raman spectrum.

This work is organized as follows. The first part is dedicated to the methodology used in this work for both the ground- and excited-state calculations. In the Results and Discussion, a detailed analysis of the TCNE:π:1ClN intermolecular CT complex in terms of structural parameters, population analysis, and vertical excitation energies is given. The electronic absorption spectrum computed from structures regularly extracted from an AIMD trajectory sampled in the ground state is also discussed in detail. Finally, the roles of two counterions in different coordination sites of the TCNE radical anion in acetonitrile solution are addressed to unravel the still-debated spectral region between 1300 and 1500 cm^–1^. The last section is dedicated to a conclusion and future perspective.

## Computational Methodology

For the TCNE:π:1ClN CT complex, we performed preliminary characterizations of both the ground state and the first singlet excited state using methods relying on a full quantum mechanical approach rooted in DFT and its time-dependent version in the linear response formalism (LR-TDDFT)^45−48^ for modeling the excited-state properties.^34−36^ Energies, unconstrained structural optimizations, and related harmonic and anharmonic^49^ frequency calculations and Raman activities for the ground electronic state were computed using the B3LYP hybrid exchange–correlation functional^50−52^ and split-valence double-ζ basis sets with diffuse functions (6-31+G(d,p)) for non-hydrogen atoms. Weak dispersion forces between the two subunits were described using correcting potentials with Grimme’s dispersion (GD3).^53−58^ Additionally, we accounted for bulk solvent effects using the polarizable continuum model in its conductor-like version (C-PCM)^59−64^ to model the solvent environment around the CT complex. Specifically, dichloromethane (DCM) (ε = 8.93) was considered as the solvent according to the experimental conditions.^23^ Excited-state energies, gradients, and infrared and Raman frequencies were computed at the CAM-B3LYP/6-31+g(d,p)/C-PCM(DCM)/GD3 level. Additionally, the S_2_ minimum-energy structure was also computed starting from the most stable ground-state conformer (in what follows, we will discuss only some relevant electronic properties rather than the structural arrangement). The choice to use two different functionals was motivated by the following considerations: the B3LYP global hybrid functional takes into account the relevant physics to predict the ground-state energetics but can be less accurate in predicting the right energy ordering of electronic transitions that involve non-negligible charge transfer and also fails to describe the correct shape of the PES along the distance coordinate between the electron donor and electron acceptor.^65,66^ On the contrary, range-separated hybrid functionals such as CAM-B3LYP are more accurate in predicting CT excitation bands and Rydberg-like excitations, as has been widely reported in the literature.^67−73^ The level of theory chosen here was previously proved to be accurate in a previous study.^32^

Free energy sampling was performed via AIMD^74−78^ in solution utilizing C-PCM for DCM. The ground-state AIMD simulation in DCM solvent was collected for 10 ps, following 1 ps of equilibration, with a time step of 0.2 fs. The atom-centered density matrix propagation (ADMP) scheme^79−86^ was employed using the smaller 6-31G(d,p) basis set, and a temperature of 300 K was enforced by rescaling the nuclear velocities each 1 ps. The electronic absorption spectrum was simulated by computing the excitation energies to the first five singlet excited states on 500 snapshots regularly extracted (each 20 fs) from the 10 ps long AIMD trajectory. Additionally, the crucial choice of the initial coordinates and momenta to run excited-state AIMD trajectories^32^ was also recently discussed in detail in ref (87) by some of the present authors. For potential calibration, vertical excitation energies to the first five singlet excited states were also evaluated on a reference structure, the ground-state minimum, also with the smaller 6-31G(d,p) basis set in both vacuum and implicit DCM solvent (see Table S1). The amount of charge separation between the two fragments in the ground state and the CT state following the excitation were quantified by natural bond orbital (NBO) population analysis^88−92^ and two indexes: the spatial distance between the barycenters of the density increment and depletion upon excitation, *d*^CT^, and the amount of charge transferred, *q*^CT^ (which for one-electron excitation ranges from 0 to 1).^93−99^ Furthermore, the ground-state coordination geometries of the TCNE doublet radical anion in the presence of a counterion (Na^+^ or K^+^) were also investigated through structural optimization and anharmonic vibrational frequency evaluations using the C-PCM to account for implicit solvation effect of acetonitrile (ACN) solvent (ε = 38.8). All of the calculations were carried out using the Gaussian 16 program suite.^100^

## Results and Discussion

### Absorption Spectrum and Structural Analysis of the S_0_ and CT States

**Figure 2:**
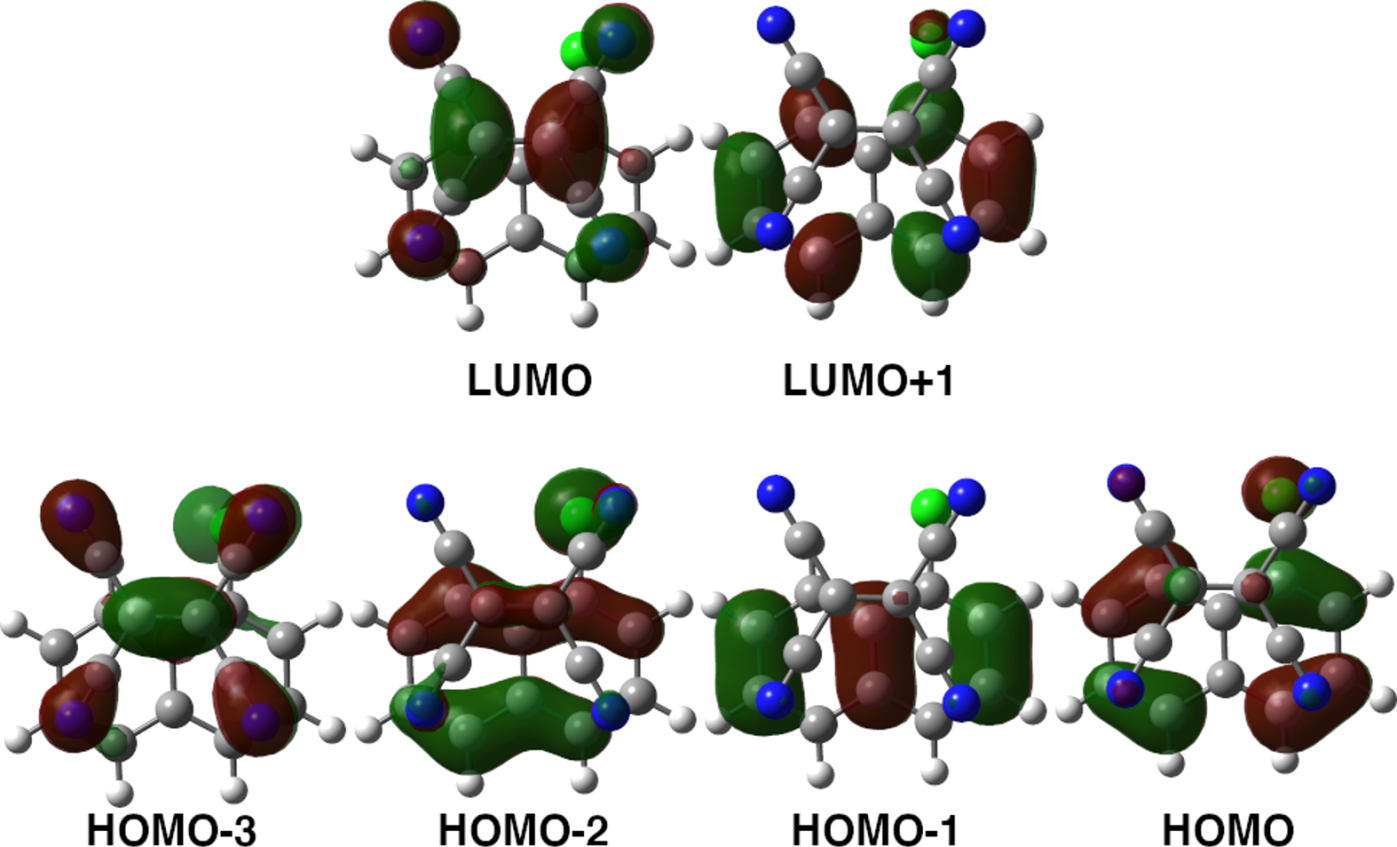
Contour plots (isovalue = 0.03) of molecular orbitals that characterize the first five excited-state electronic transitions: (bottom) occupied molecular orbitals HOMO–3 to HOMO and (top) unoccupied molecular orbitals LUMO and LUMO+1, computed at the CAM-B3LYP/6-31+g(d,p)/C-PCM(DCM)/GD3 level of theory.

The vertical excitation energies were computed from the ground-state minimum-energy geometries (discussed in detail below; see Table 1), for comparison with the known experimental UV–vis electronic absorption spectrum of TCNE:π:1ClN CT complex in DCM. The experimental optical spectrum profile in solution shows two distinct absorption bands with maxima centered at 408 nm (3.04 eV) and 537 nm (2.31 eV).^23^ In detail, we found that the less energetic transition (S_1_ ← S_0_) has HOMO–LUMO character (see Figure 2), in which the HOMO is completely localized on the donor monomer and the LUMO is spatially confined on the TCNE molecule, with a high transition dipole moment (TDM) of 2.33 au. The excited-state dipole moment is significantly larger than the ground-state one (see Table 2), confirming the CT character of the transition. In particular, for this excited state we also evaluated the *d*^CT^ and *q*^CT^ indexes reported in Tables 2 and S3 to estimate both the spatial charge transfer distance and the charge transferred upon excitation, respectively. The less intense S_2_ ← S_0_ transition (TDM = 0.005 au), which has CT character, mainly involves the HOMO–1, localized on the naphthalene core of the 1ClN unit, and the LUMO of TCNE, as clearly reported in Figure 2. Furthermore, the significant increase in the dipole moment computed following the nuclear relaxation on the S_2_ electronic state (13.22 D) with respect to the ground-state one corroborates the CT nature of the electronic transition.

**Table 1:** Vertical Excitation Energies (in eV) and (in Parentheses) Oscillator Strengths (*f*) for the Three TCNE:π:1ClN CT Complexes in DCM Solvent Computed Using the LR Formalism; Experimental Values (in eV) Observed under the Same Conditions^23^ and Δ(S_2_–S_1_) Values (in eV) Are Also Reported

	Exptl	Min_S_0_a_	Min_S_0_b_	Min_S_0_c_
S_1_ ← S_0_ VEE (*f*)	2.34	2.20 (0.1251)	2.13 (0.1108)	2.18 (0.0989)
S_2_ ← S_0_ VEE (*f*)	3.04	2.95 (0.0004)	2.88 (0.0028)	2.95 (0.0064)
Δ*S*_2_–S_1_	0.70	0.75	0.75	0.77

**Table 2:** Comparison of NBO Total Charges *q*_NBO_ (in *e*) and Dipole Moments μ (in D) Calculated in Implicit DCM Solvent for the TCNE:π:1ClN CT Complex in the Ground State (S_0_) and First Singlet Excited State (S_1_); The Center-of-Mass Distances *d*_CoM_ (in Å), CT Distances *d*^CT^ (in Å), and Transferred Charges *q*^CT^ (in *e*) Are Also Given

Parameter	S_0_	S_1_
*q*_NBO_	±0.083	±0.840
μ	3.92	14.95
*d*_CoM_	3.534	3.469
*d*^CT^, *q*^CT^	–	2.506, 0.968

Initial test calculations, reported in Table S1, were performed to evaluate the effects of the basis set size and the role of the dichloromethane bulk solvent at C-PCM level on the first five electronic transitions. We found that for all cases considered the absolute error with respect to the experimental value for both transitions was less than 0.1 eV, but more importantly, the energy gap between the electronic states is well-reproduced. The inclusion of the implicit DCM solvent in the model systematically led to a red shift of the vertical excitation energies, in particular about −0.07 eV for the first two transitions obtained at the CAM-B3LYP/6-31G(d,p) level. Considering the effect of a larger basis set with diffuse functions for non-hydrogen atoms, in gas phase there is a red shift at lower energies of ∼0.07 eV (Δ = −0.08 eV for S_1_ and −0.06 eV for S_2_). When implicit solvent is also taken into account in the model, a trend similar to the smaller basis set is observed (−0.07 eV compared to those computed in the gas phase). Interestingly, there is a slight increase of 0.03 in the oscillator strength (*f*) associated with each electronic transition for both basis sets when the implicit solvent is included in the modeling, making the S_1_ transition brighter. In summary, the long-range-corrected Coulomb attenuated hybrid functional CAM-B3LYP in conjunction with both basis sets considered proved to be reliable in predicting the vertical excitation energies and their CT character. Our findings confirm that the addition of diffuse functions on non-hydrogen atoms is particularly indicated for modeling of charged systems in the excited states and for the affordable description of charge transfer and spatially delocalized excitations as valence-Rydberg, doubly excited, and ππ*.^101−104^ Finally, we chose to include the implicit solvation model to take into account the electrostatic contribution of the solute–solvent interaction to describe the model system in a more realistic way. Relying on the accuracy of the level of theory, we proceeded to analyze the CT next.

According to Mulliken theory^105,106^ π-stacked molecular complexes such TCNE:π:1ClN arise from an Lewis acid–base interaction and can be described in terms of resonance between a neutral D−π–A structure and a dative D^+^–π–A^–^ structure. On the contrary, in the past years the studies of Morokuma,^107^ Lippert,^108^ and Roeggen^109^ have shed light on the importance of different types of intermolecular forces (i.e., polarization, electrostatic, charge transfer resonance, and van der Waals), which are responsible for the stabilization of the weakly bound electron D–A complexes.^110^ Actually, due to weak interactions, a fair charge transfer of electron density is already observed between the two units in the ground state, in which the low vibrational levels are populated at the thermal energy. On the other hand, electromagnetic radiation is capable of photoinducing a drastic reorganization of the electron density in the whole molecular system, leading to the formation of an ion pair. For this purpose, we focused on the relative orientation of the two monomers, the solvent effect, vibrational frequency shifts, and the distance between the molecular planes in both the ground and excited electronic states, since their different electron density (re)distributions in the complex can play a key role in the CT event and need to be carefully investigated. Consequently, due to the non-covalent nature of the complex under investigation, it is reasonable to expect that there are several ground-state conformers representative of the equilibrium ensemble at room temperature, and indeed, in this work different cofacial starting configurations of the TCNE:π:1ClN dimer were chosen to model the relative orientations assumed by the two subunits. Initial geometries for the geometry optimizations were chosen by starting from three different TCNE:π:1ClN configurations leading to different minima (denoted as Min_S_0_a_, Min_S_0_b_, and Min_S_0_c_, as reported in Figures S1 and S2) in the ground electronic state with negligible energy differences (less than 1 kcal/mol). All of the conformers were fully planar, retaining the π–π-stacked arrangement. In Min_S_0_a_, the TCNE unit lies on the ring bearing the chlorine atom with the C=C bond parallel to the underlying C–Cl bond. For the more energetic Min_S_0_b_, the two monomers are in a distorted orthogonal conformation in which TCNE is above the two bridghead carbons at a greater distance from 1ClN (∼0.25 Å) compared to those observed for the other two conformers. The arrangement of Min_S_0_c_ structure is almost specular to the Min_S_0_a_ one, although slightly distorted and 0.51 kcal/mol more stable than the previous one. A summary of vertical excitation energies (VEEs) computed within the LR-TDDFT formalism for all of the conformers is reported in Table 1, and the frontier molecular orbitals (MOs) involved in the CT transitions are shown in Figure S5.

**Figure 3:**
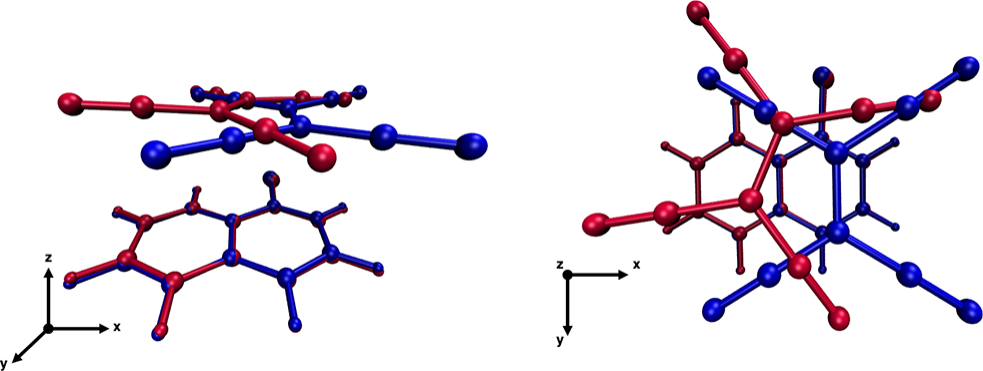
Front (left) and vertical (right) views of Min_S_0_a_ (blue) and Min_S_1_a_ (red) of the TCNE:π:1ClN CT complex in implicit DCM solvent.

From gradient minimization procedures on the PES for the first singlet excited state (S_1_), three stationary points were found. The relative positions of the two subunits for two of the minima (Min_S_1_a_ and Min_S_1_c_) were very similar in the spatial arrangement and in terms of relative energy content. In the Min_S_1_b_ stationary point, the TCNE and 1ClN monomers were superimposed on each other at their centers perpendicularly. We will focus our discussion mainly on only a single pair of minima, one for the ground state (Min_S_0_a_) and one for the excited electronic state (Min_S_1_a_), reported in Figure 3.

We find that in the ground state the TCNE subunit lies in parallel to the donor ring bearing the chlorine substituent. From a geometrical point of view, the central C=C bond length calculated for TCNE is 1.378 Å, which is 0.030 Å (0.040 Å) longer than the related values in isolated neutral TCNE (neutral TCNE:π:donor complexes).^111,112^ This implies that at equilibrium the weak non-covalent dispersive interaction forces with 1ClN and the low polarity of the DCM solvent do not significantly modify the TCNE structure considering that the C=C double bond length (and related bond order) is an important probe with respect to the electron density rearrangement. The carbon atoms bridged with the cyano groups are at an average distance of 1.428 Å, whereas the C–N distance is 1.163 Å, consistent with a typical triple bond. The 1-chloronaphthalene molecular structure in the adduct is in nice agreement with the X-ray diffraction method experiments in the liquid phase,^113,114^ within 2% error for bond lengths, except for the C–Cl distance, which is different by 0.05 Å. In the optimized structure of the TCNE:π:1ClN complex there is a weak charge transfer between the two units (less than 0.1e considering the NBO population analysis; see Tables 2 and S3) that contributes to stabilization of the adduct. Both monomers retain a highly planar structure: for TCNE the N_1(2)_C=CN_3(4)_ dihedral angles are almost 0°, as are the two dihedral angles of the 1ClN donor (0.02° for C_1_–C_6_–C_8_–C_9_ and 0.07° for C_1_–C_6_–C_4_–C_3_), and the computed distance between the two subunits considering their respective centers of mass is 3.534 Å. For the other conformers considered, the same conclusions are reached, as they showed strictly similar trends and are reported in Figure S1.

**Figure 4:**
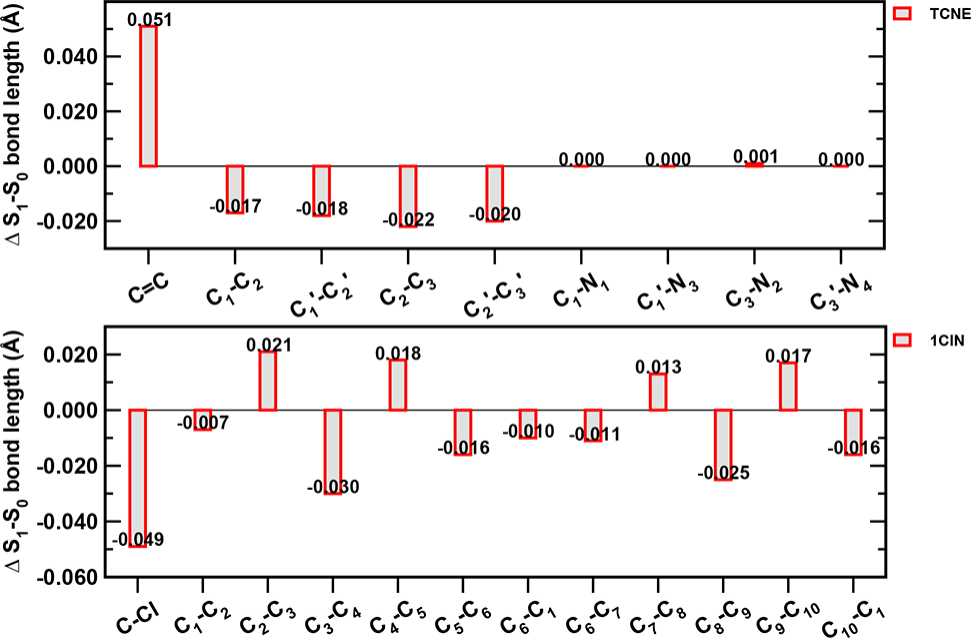
Bar chart showing the difference between the bond lengths (in Å) for each monomer (top, TCNE; bottom, 1ClN) calculated between the ground state and first singlet excited state minimum-energy structures (Min_S_0_a_ and Min_S_1_a_, respectively). The labeling scheme is shown in Figure 1.

Following the photoexcitation and subsequent relaxation on the S_1_ excited-state PES, the sudden electron density reorganization induces relevant forces and structural rearrangements (see Figure 4). The 1ClN monomer exerts an electron donor role by transferring a non-negligible amount of electron density to the TCNE, as can be inferred from the NBO population analysis (±0.840e; Table 2), and indeed, experimentally the presence of a biradical couple has been hypothesized.^23^ The electron density rearrangement associated with the S_1_ ← S_0_ electronic transition is reported for the three minima for comparison in Figure S6. The center-of-mass distance between the two monomers decreases significantly with respect to the neutral ground state because of the photoinduced charge distribution, and the two monomers are closer in space (see Table 2), as observed in a similar electron donor–acceptor complex (TCNE:hexamethylbenzene^115^). The extent of the intermolecular charge transfer was also evaluated for the first singlet excited state through calculations of the *d*^CT^ and *q*^CT^ indexes (Tables 2 and S3), which help to quantify the spatial extent and the overall amount of charge transferred upon excitation, respectively. We found that the *d*^CT^ values are similar for the three orientational isomers considered and that the *q*^CT^ values are maximum when the TCNE monomer is closer to the unsubstituted ring of the donor, as in the Min_S_0_b_ and Min_S_0_c_ cases. With regard to the TCNE acceptor monomer, the initially planar structure becomes slightly distorted: the four substituents of the double bond are slightly off the plane (N_1_C=CN_3_ = −5.06°, N_2_C=CN_4_ = −6.56°), and the central C=C double bond undergoes an elongation of 0.051 Å. On the contrary, the carbon atoms bridged with the cyano groups are at an average distance of 1.409 Å. The four CN triple bonds seem to be unaffected by photoexcitation, as they do not undergo any variation. The molecular geometry of 1ClN is still planar, but several structural rearrangements occur following the photon absorption. Mainly the chlorine atom at the α-position of the naphthalene unit exerts resonance-donating effects that push electron density onto the naphthalene rings, resulting in a shortening of the C–Cl bond length by 0.05 Å and reorganization of all of the C–C bonds. In particular, the bridging bond (C_1_–C_6_) and its nearest neighbors (C_1_–C_10_, C_1_–C_2_, C_6_–C_7_, and C_5_–C_6_) are shortened by ∼0.01 Å, as are C_3_–C_4_ and C_8_–C_9_ to a greater extent (∼0.03 Å). The remaining C–C bonds relax, stretching by ∼0.02 Å. In Figure 4 we report a resume of the main structural changes discussed above that occur following the photoexcitation.

Molecular systems ruled by weak dispersion interactions can explore a large conformational space, visiting numerous stationary points, overcoming low energy barriers, or even proceeding via barrierless conversions. The potential energy hypersurface of a non-covalent molecular complex is then very challenging to describe accurately, and we expect it to contain a large number of almost degenerate energy minima for which the definition of a representative equilibrium becomes very challenging. Ab initio molecular dynamics simulations provide a wealth of deeper insight that can be pulled out by subtly analyzing the trajectories, which can help in the prediction or interpretation of the experimental data at an atomistic level. To this end, we also performed an extensive molecular dynamics study^32^ of the TCNE:π:1ClN CT complex in DCM, characterizing the PESs of the ground and excited states at finite temperature, refining the vibrational frequency assignments, and also identifying the excited-state vibrational modes that mainly contribute to the nuclear relaxation from the Franck–Condon region toward the ground state. From the visual inspection of the ground-state AIMD trajectory, the face-to-face arrangement of the two subunits is always retained. We report in Figures S3 and S4 the normalized distributions of selected structural parameters for the TCNE and 1ClN monomers, respectively, obtained from the ground-state AIMD trajectory. The central double bond distribution of TCNE (Figure S3A) is peaked at 1.377 Å, in good agreement with the minimum-energy structures characterized in this work (also see Table S3). Similar considerations apply to the C≡N bond length distributions centered at 1.162 Å (Figure S3B) and the N–C–N bond angle distributions (Figure S3C). The TCNE in the ground-state potential well is a highly planar molecule. The distributions of both N–C=C–N dihedral angles computed from the 10 ps long AIMD trajectory (Figure S3D) cover a tight range of values (ca. 40°), and are both centered at 180°, suggesting that there are no structural distortions during the PES exploration. The C–Cl bond length is 1.760 Å on average for the three representative minima considered. In Figure S4A the bond distribution covers a range of ∼0.25 Å showing two peaks at 1.740 and 1.780 Å reflecting small fluctuations around the average value. The distributions of the central ring C–C bonds (Figure S4B) overlap well with the analogous minimum-energy structure parameters, which means that in the ground state the formation of the adduct in DCM solution does not alter the aromatic structure of the donor monomer. In Figure S4C we report the distribution of the center-of-mass distance between the two subunits. In addition to the main peak at 3.50 Å, we observe two less populated distances, namely, ∼3.76 and 4.30 Å. These results prove the presence of different mutual arrangements explored during the AIMD simulation resulting from the weak Coulombic interactions that govern the π-stacking arrangement and allow the TCNE to slide on the naphthalene core. Therefore, the three structures we have selected (Figure S1, left column) are representative of the ground-state PES, which appears to be flat as expected for a non-covalent complexes. Finally, the distribution reported in Figure S4D accounts for the dihedral angles between the two condensed rings of the 1-chloronaphthalene. From the comparison with the same parameters measured for Min_S_0_a_, in the ground state there are no remarkable deviations from planarity.

### Optical Absorption of TCNE:π:1ClN in DCM Solution

**Figure 5:**
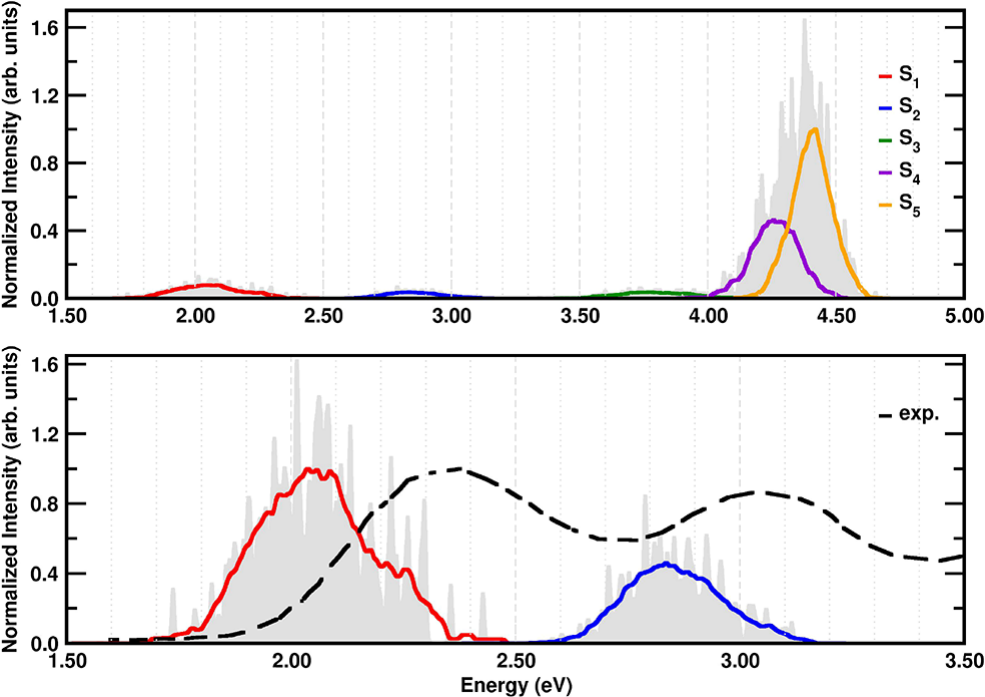
(top) Absorption spectrum of the TCNE:π:1ClN complex computed at room temperature in implicit DCM solvent at the TD-CAM-B3LYP/6-31+g(d,p)/C-PCM(DCM) theory level. (bottom) Focus on the S_1_ ← S_0_ and S_2_ ← S_0_ CT bands investigated in this work and the experimental absorption spectrum (dashed black line) retrieved from ref (23). Intensity is in arbitrary units and normalized. Colored lines represent averages computed over 200 points. The color scheme is reported in the legend.

The experimental absorption spectrum recorded in DCM solvent, reported in ref (23), shows two bands in the 380–700 nm range with maxima at 537 nm (2.31 eV) and 408 (3.04 eV), corresponding to electronic transitions from two π orbitals of 1ClN with CT character. The simulated absorption spectrum of the TCNE:π:1ClN complex in implicit DCM solvent at room temperature covers the entire UV–vis spectral region and is shown in the top panel of Figure 5. In the 800–260 nm region, five electronic excitations with different relative intensities are present. At low energy, two bands centered at 2.05 and 2.83 eV are visible that have HOMO–LUMO and HOMO–1–LUMO character, respectively (see Figure 2). The maximum of the first CT band is red-shifted from the experimental value by 0.26 eV, and there is a similar but smaller shift for the second CT transition, for which the agreement is slightly better (0.21 eV). The relative intensities of the two bands just discussed (red and blue lines) are reproduced well in comparison with the experimental data (see the bottom panel of Figure 5). At higher energy, an additional weak CT band (green) is centered at 3.79 eV, and in this case the electronic transition is mainly dominated by the HOMO–2 orbital localized on the 1ClN donor and the LUMO confined on the TCNE acceptor. The most intense band is in the blue region of the optical spectrum, peaked at 4.37 eV. Two different electronic transitions that are close in energy contribute to this feature, namely, the S_4_ ← S_0_ and S_5_ ← S_0_ transitions. The analysis of the molecular orbitals mainly involved reveals that the nature of the excitation is of the local type. In particular, in S_4_ (violet) contributions from local excitations in both subunits are present (HOMO–3–LUMO for TCNE and HOMO–LUMO+1 for 1ClN; see Figure 2). On the contrary, S_5_ (orange) is dominated by local excitations of 1ClN with a HOMO–1–LUMO+1 and HOMO–LUMO+1 mixed nature. It is worth noticing that this system shows a very dense electronic manifold, where the interplay between vibrations and electronic states can rule the relaxation dynamics after the photoexitation.

### Ground- and Excited-State Raman Vibrational Fingerprints

**Figure 6:**
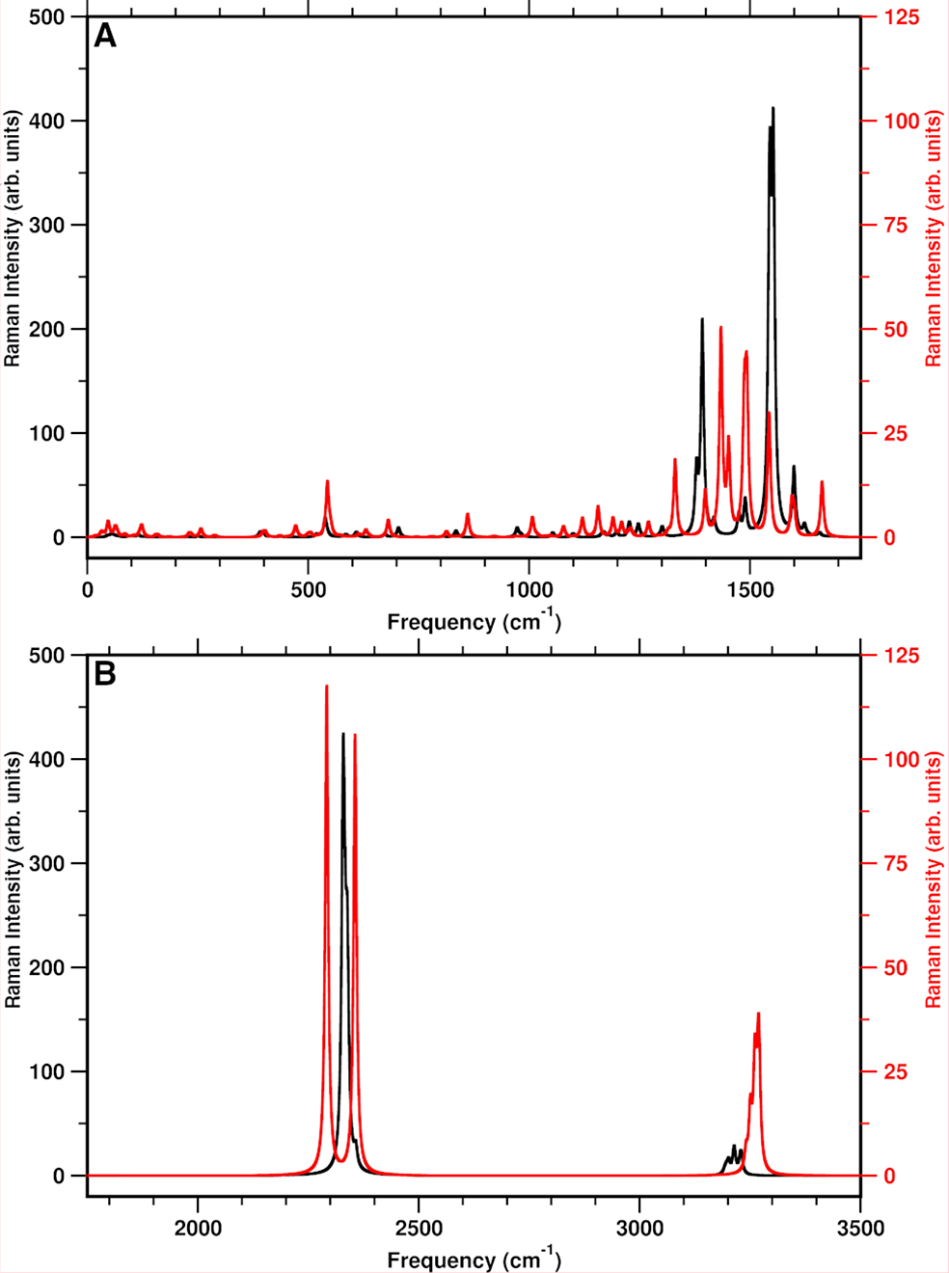
Vibrational Raman spectra of the ground state (black) and first singlet excited state (red) (intensities are in arbitrary units with a half-width at half-height of 4 cm^–1^) of the TCNE:π:1ClN CT complex in implicit DCM solvent computed at the B3LYP/6-31+G(d,p)/C-PCM(DCM)/GD3 and TD-CAM-B3LYP/6-31+G(d,p)/C-PCM(DCM)/GD3 levels, respectively. For clarity, the 0–1750 cm^–1^ spectral range is reported in (A) and the 1750–3500 cm^–1^ spectral range in (B).

In Figure 6 we report the harmonic Raman spectra of the TCNE:π:1ClN CT complex in DCM solvent computed in the S_0_ and S_1_ electronic states to have a clear picture of the vibrational normal mode composition and related frequencies mainly affected by photoexcitation. At first glance the most intense Raman features are present in three distinct spectral regions: 1400–1700 cm^–1^, 2200–2400 cm^–1^, and around 3200 cm^–1^. Before the discussion of the vibrational fingerprint analysis, it should be noted that the vibrational bands of the photoexcited complex observed in the time-resolved Raman vibrational spectrum, mainly attributed to the acceptor monomer, were unraveled by relying on the different vibrational signatures of the TCNE radical anion in solution and crystalline phase. In particular, the splitting of the vibrational band (at 1390 and 1420 cm^–1^) resulting from the central C=C stretching mode of chemically reduced TCNE in solution was tentatively assigned assuming that the counterion was inside or outside the solvation shell.

The first vibrational frequency with a non-negligible Raman intensity in S_0_ is peaked at 1379 cm^–1^ and arises mainly from the C1–C2 and C4–C5 asymmetric stretching motion and the in-plane bending mode of the HCCH pattern of the 1ClN monomer. In the S_1_ excited state this mode is blue-shifted by 20 cm^–1^, preserving the mode composition. The asymmetric ring stretching mode (where C1–C6, C7–C8, and C9–C10 mainly contribute) is centered at 1392 cm^–1^ in the ground state and is blue-shifted by 42 cm^–1^ to 1434 cm^–1^ in the S_1_ excited state. The two intense Raman features centered at 1545 and 1553 cm^–1^ originate from the central C=C double bond stretching mode of the TCNE electron acceptor coupled to different C–C stretching modes located at the end of the donor monomer (mainly C3–C4 and C8–C9). Upon excitation, this mode becomes essentially localized on the TCNE monomer and undergoes a remarkable red shift of ∼60 cm^–1^ to 1487 cm^–1^. To a lesser extent, the TCNE C=C stretching is also involved in the vibrational mode composition of the 1600 cm^–1^ band, in which the C1–C6, C3–C4, and C8–C9 atom pairs contribute largely in synchronous stretching. In the S_1_ excited state, the new electronic rearrangement induces a noticeable blue shift of 64 cm^–1^, as expected by inspection of the MOs (see Figure 2), and the TCNE unit does not contribute to the mode. The spectral region around 2300 cm^–1^ is populated by the two symmetric and two asymmetric stretching modes of the CN triple bonds, which in the ground state are found to be almost degenerate. The symmetric stretching modes of all CN groups are centered at 2329 and 2357 cm^–1^ in S_0_, and in S_1_ the first one is blue-shifted by less than 30 cm^–1^ (to 2356 cm^–1^) and the other one is only weakly affected by photoexcitation (2348 cm^–1^). The asymmetric CN stretching modes are separated by 5 cm^–1^ (2333 and 2338 cm^–1^), and both undergo a decrease in frequency in the S_1_ excited state by 41 and 37 cm^–1^, respectively. Finally, all of the remaining C–H stretching modes of the 1ClN donor monomer are blue-shifted on average by ∼45 cm^–1^ with respect to the ground-state ones. This specific dimer was recently investigated^23^ in DCM solution by means of spontaneous Raman spectroscopy and femtosecond stimulated Raman spectroscopy (FSRS), and the results presented in this work are in good agreement with those experimental findings (see Table 3 for a comparison), except for the C=C double bond stretching mode of the TCNE acceptor (exptl 1392 cm^–1^). The out-of-plane bending mode, localized on the TCNE monomer, is at 156 cm^–1^ in the ground state and is almost unaffected upon photoexcitation (153 cm^–1^). In the S_1_ excited state, the feature at 534 cm^–1^ has been assigned to the CCN symmetric in-plane bending. The agreement has been also verified in our recent work for the two low-frequency modes, and the same mismatch has been found regarding the C=C stretching mode.^32^ A detailed discussion about the central C=C stretching mode of the TCNE unit is given in the following section.

**Table 3:** Harmonic and Anharmonic Frequencies ω (in cm^–1^) Calculated in the Ground State (Min_S_0_a_) and Harmonic Frequencies Computed in the First Excited State (Min_S_1_a_) for the CT Complexes in Implicit DCM Solution in Comparison with Experimental Values Found in the Literature

	S_0_			S_1_	
normal mode	ω_harm_	ω_anh_	Δω	ω_harm_	others
TCNE out-of-plane bending	165.93	165.54	–0.39	159.14	168,^a^ 165,^b^ 156,^c^ 153^c^
symmetric in-plane CCN bending	538.17	575.41	37.24	550.79	542,^b^ 534^c^
ν_C=C_	1552.62	1594.35	41.73	1486.86	1392,^c^ 1565,^c^^,^^d^ 1570,^d^ 1551,^e^ 1421^f^
ν_C≡N_ symm	2329.15	2284.27	–44.88	2356.16	
ν_C≡N_ symm	2357.76	2319.04	–38.72	2291.80	
ν_C≡N_ asymm	2333.06	2301.28	–31.78	2347.74	
ν_C≡N_ asymm	2338.50	2301.28	–37.22	2300.90	
ν_C–Cl_	973.00	960.45	–12.56	1007.41	953^c^
ν_C1–C2_	1378.70	1354.94	–23.76	1398.60	1362^c^

a TCNE:hexamethylbenzene resonance Raman excitation in DCM, from ref (115).

b TCNE:hexamethylbenzene resonance Raman excitation in CCl_4_, from ref (116).

c TCNE:1-chloronaphthalene spontaneous Raman and FSRS spectra in DCM, from ref (23).

d Ground-state neutral and complexed TCNE, from ref (117).

e TCNE:hexamethylbenzene resonance Raman excitation in CCl_4_, from ref (118).

f TCNE radical ion, from ref (119).

In Table 3 we report a summary of harmonic and anharmonic frequencies computed in the ground state, the harmonic frequencies calculated for the first singlet excited state, and other theoretical and experimental counterparts when available. The Min_S_0_a_ and Min_S_1_a_ stationary points were chosen as reference structures. The normal mode displacement vectors of Table 3 are reported in Figure S7. As in the previous analyses, it is worth highlighting that this system shows a very dense vibrational manifold that is influenced by the electronic potential, where the interplay between vibrations and electronic states can definitively dictate the relaxation dynamics after the photoexcitation.

### Structures and Vibrational Fingerprints of TCNE:M^+^

**Figure 7:**
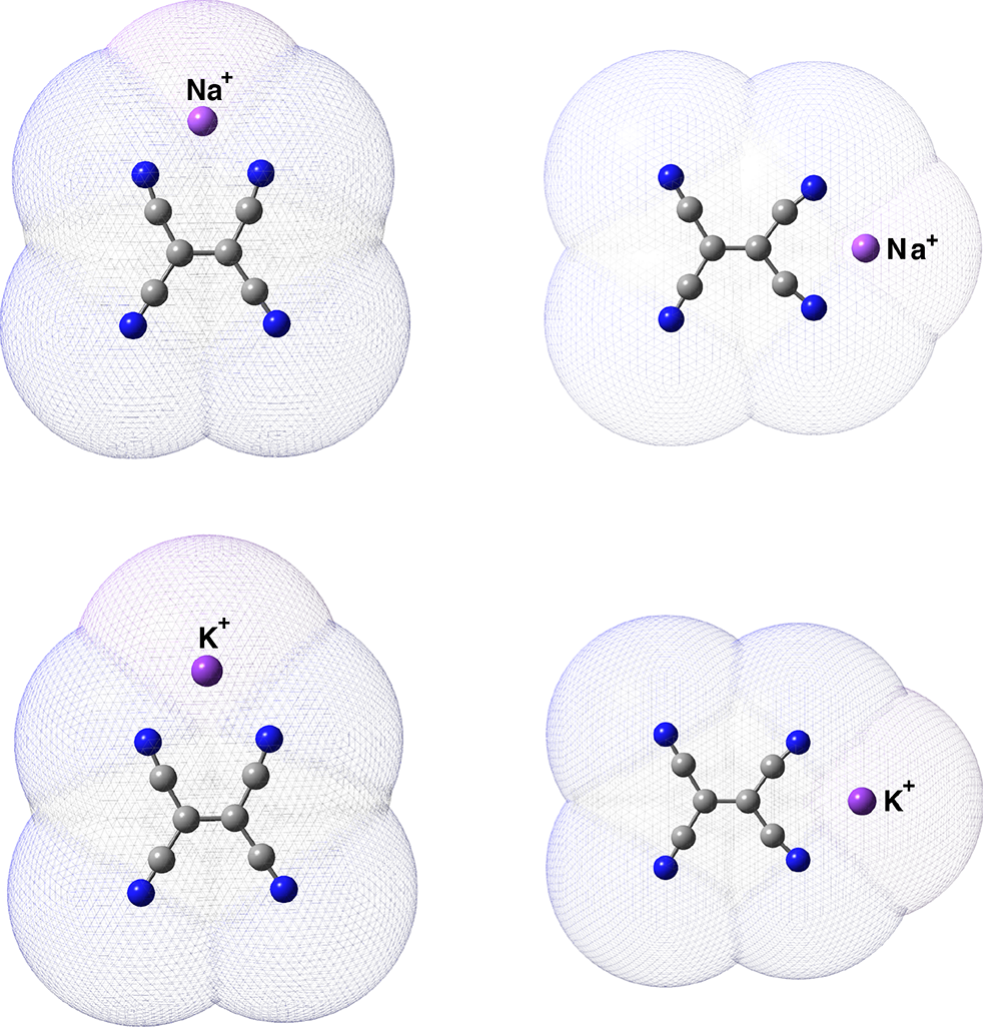
Two different coordination sites of TCNE ^•–^ with Na^+^ and K^+^ countercations in the top and bottom rows, respectively. The C-PCM acetonitrile solvent-accessible surface cavity is also shown. Left: the counterion is in the C–C=C–C major groove. Right: the counterion is in the C–C–C minor groove. Main structural parameters are reported in Figure S8.

**Figure 8:**
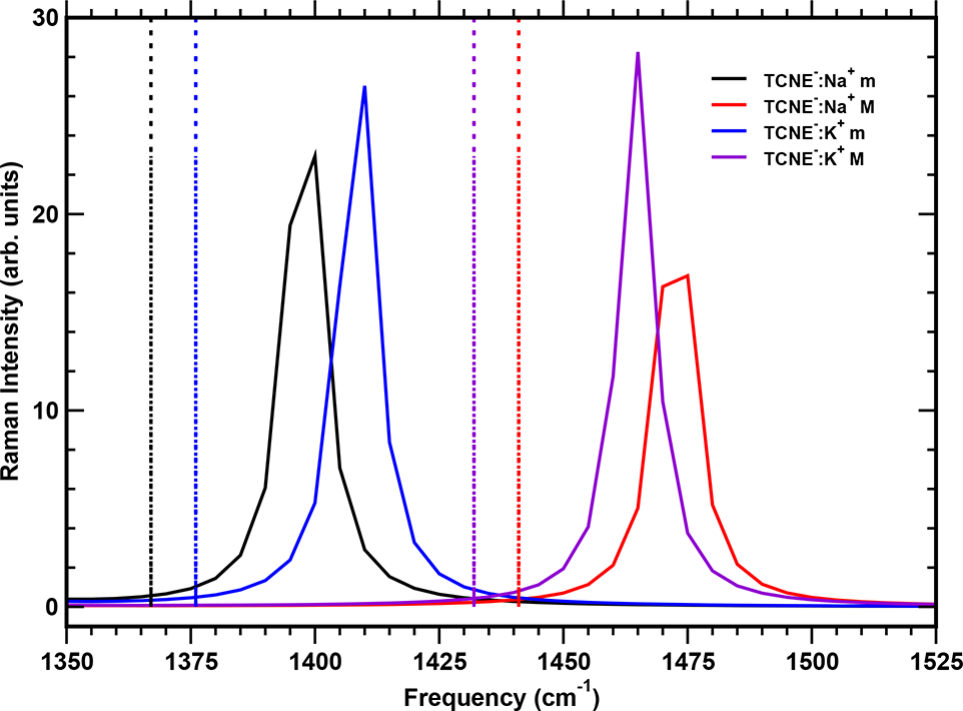
Ground-state harmonic Raman spectra (solid lines) of the TCNE^•–^:Na^+^ and TCNE^•–^:K^+^ complexes computed in implicit acetonitrile solvent in the 1350–1525 cm^–1^ spectral region. The minor and major coordination sites are labeled as m and M, respectively. Corresponding anharmonic values are reported as colored dashed lines. The inset shows the color scheme adopted.

From FSRS experiments conducted on the TCNE:1ClN CT complex in dichloromethane solution, Mathies and co-workers^23^ assigned the excited-state C=C stretching mode of TCNE to 1392 cm^–1^, taking into account that the ground-state vibrational spectra of TCNE chemically reduced with NaI or KI in acetonitrile solution showed two peaks at 1390 and 1421 cm^–1^ and hypothesizing that these peaks were due to the C=C stretching of TCNE^•–^ for which the countercation was *inside* or *outside* the solvation shell. We theoretically investigated also the possible coordination sites of the Na^+^ or K^+^ counterion on TCNE in implicit ACN solvent in accordance with the experimental conditions (Figure 7) to elucidate the main structural and vibrational frequency changes of the central C=C and C≡N bonds when coordinated together. Crystallographic structures containing anionic TCNE moieties in monomeric TCNE^•–^ form and as dianionic (TCNE)_2_^2–^ π dimers are known, and some other computational and/or spectroscopic studies have been performed both in the solid phase and in vacuum,^120−123^ but to the best of our knowledge a detailed investigation of the monomeric radical anion coordination sites in solution is still absent. The structural guess for the TCNE:M^+^ pairs was built taking into account first the *D*_2*h*_ planar structure of the TCNE unit and the fact that two distinct C=C and symmetrical C≡N stretching frequencies were observed experimentally in solution for each species, making us assume that the counterion was arranged according to two symmetrical positions with respect to the TCNE unit. The TCNE:M^+^ pairs were modeled considering at least three different coordination sites: the C–C=C–C major groove and the C–C–C minor groove both in the molecular plane and on the top of the carbon double bond, which have never been isolated. Upon the geometry optimization process, we found two stable geometries that correspond to two different coordination sites on the TCNE molecule. For all cases considered, the TCNE molecule assumes a distorted *D*_2*h*_ planar structure, and the proximity of the countercation strongly polarizes the central C=C bond, which in the ground-state minimum-energy structure showed a distances of 1.448 (1.433) Å for the Na^+^ ion and 1.446 (1.434) Å for the TCNE^•–^:K^+^ complex when the ion was in the C–C=C–C major (minor) groove (we recall that for the optimized TCNE monomer in the ground electronic state in implicit DCM solvent, this distance was equal to 1.371 Å, and the anharmonic C=C stretching frequency was 1548 cm^–1^). The bond lengths of the CN groups directly involved in the interaction with the counterion undergo a slight elongation (from 1.169 to 1.174 Å, on average). When the positive species is in the minor groove, the exposed NCN angle (at 120° under isolated conditions) is significantly reduced by ∼26° and ∼21° for Na^+^ and K^+^, respectively. The labeling schemes of the structural parameters just described are reported in Figure S8. The two different coordination sites are found also to be responsible for two distinct values of the computed Raman anharmonic stretching frequencies associated with the C=C stretching mode: 1367 (1376) cm^–1^ for TCNE^•–^:Na^+^(K^+^) in the minor groove and 1441 (1432) cm^–1^ for the TCNE^•–^:Na^+^(K^+^) complex in the other case (see Figure 8). The frequency red shifts in fairly good agreement with the experimental infrared spectra of the TCNE^•–^:Na^+^/K^+^ anion salts^117,124,125^ can be also rationalized in light of the difference in the experimentally measured electron affinities of the cations, which we took into account in the calculations (0.046 eV greater for Na than for K (0.501 eV)).^126,127^ We found two CN symmetric stretching modes each for the two coordination sites considered: 2151/2207 (2181/2204) cm^–1^ for K^+^ in the major (minor) groove of TCNE^•–^ and 2149/2208 (2172/2204) cm^–1^ for Na^+^ in the major (minor) groove of TCNE^•–^ (the lower values concern the CN stretching of the cyano groups directly facing the counterion), in agreement with tetracyanoethylene anion radical resonance Raman measurements by Van Duyne and co-workers.^117^ It is interesting to note that regardless of the counterion, the CN symmetric stretching mode composition changes according to the coordination site, i.e., when the cation occupies the major groove, all of the cyano groups participate in the composition of the mode, whereas in the second case the vibrational mode turns out to be composed of only one pair at a time, becoming more decoupled. From our investigations of the TCNE radical anion in solution, we can state that the two vibrational frequencies experimentally observed in the C=C spectral region are due to different coordination sites of the counterion in the same solvation sphere of the TCNE radical anion.

To summarize, we find that the vibrational feature at 1392 cm^–1^ in the FSRS spectrum can be ascribed to a specific C–C stretching mode of the donor monomer 1ClN (mainly localized on the C1–C2 bond; see Table 3 and Figure S5), while the C=C stretching mode of the photoexcited TCNE is located at ∼1487 cm^–1^. Our recent AIMD simulation study^32^ has also confirmed this assignment. The splitting observed for the vibrational band resulting from the central C=C stretching mode of the chemically reduced TCNE in ACN can be explained by considering two contact ion pairs mainly present in solution.

## Summary and Conclusions

In our work, a detailed theoretical analysis of a challenging non-covalent complex in solution has been performed using methods rooted in density functional theory. We evaluated the major structural changes of the complexes following the transition between electronic states and how the vibrational fingerprints are affected by the electron density rearrangement upon photoexcitation. The first two electronic transitions that we mainly addressed, experimentally observed for the TCNE:π:1ClN system in DCM solution at 530 and 408 nm, show charge transfer character in which the HOMO–LUMO and HOMO–1–LUMO frontier molecular orbitals are mainly involved, respectively, and are in satisfactory agreement with the experimental counterparts. From a careful investigation of representative structures found by exploring both the ground and first singlet excited state PESs, it has been possible to deduce that in solution the 1ClN donor and the TCNE acceptor monomers are arranged face to face, forming a sandwich-type structure. From the comparison with the distributions of structural parameters calculated from the AIMD trajectory in the ground state, an excellent match is observed for the representative minimum-energy structures considered in this work. Moreover, in the ground electronic state there is a very weak charge transfer from the 1ClN unit to the TCNE unit, which then increases significantly following the excitation at 530 nm and subsequent relaxation on the S_1_ state. For the photoexcited complexes the charge separation is close to unity, and the distance between the molecular planes decreases due to the greater Coulombic attraction. We also performed a careful vibrational analysis of the stationary points on the ground state and first singlet excited state to support and refine the recent findings from time-resolved spectroscopies and to have a clear picture of the vibrational normal mode composition and related frequencies mainly affected by photoexcitation. We found that the electron density reorganization upon photoexcitation and relaxation on the first excited state lead to a different vibrational mode composition and frequency with respect to thermal equilibrium. We were able to assign the experimentally recorded vibrational bands with greater confidence. According to what was observed in this work, the excited-state vibrational modes localized on the donor monomer fall in a spectral region that is wide enough to make the assignment of a vibrational mode of the TCNE probe of the charge transfer event uncertain. In particular, having the possibility of identifying in greater detail the vibrational signatures present in the spectrum, the excited-state vibrational band experimentally located at 1392 cm^–1^ has been ascribed to a specific C–C stretching mode of the naphthalene moiety, and the band at 1487 cm^–1^ has been assigned to a TCNE C=C stretching mode. Such findings were further confirmed by our recent work.^32^ Finally, an in-depth study of the coordination sites of two cationic species toward the TCNE radical anion helped on the one hand to understand that two different contact ion pairs are generated in solution and on the other to clarify the origin of vibrational features present at 1390 and 1421 cm^–1^ in the measured Raman spectrum in the spectral range typical of the C=C stretching modes. We found that when the counterion is coordinated in the major groove, the double bond is less polarized and the C=C stretching is located at 1441 (1432) cm^–1^ for the TCNE^•–^:Na^+^(K^+^) case, whereas on the contrary, when the cations lie along the C=C internuclear axis, a strong red shift is observed, to 1367 (1376) cm^–1^ for TCNE^•–^:Na^+^(K^+^). The presented analyses show that this system has a very dense vibrational manifold that is influenced by the electronic potential, where the interplay between vibrations and electronic states can definitively dictate the relaxation dynamics after the photoexcitation. Finally, this work highlights that accurate theoretical modeling of molecular systems can provide the appropriate understanding at an atomistic level of nuclear arrangements, light–matter interactions, and eventually the solute–solvent interactions that can help in fine-tuning of the optical properties of charge transfer materials useful for future technological applications.

## Supporting Information Available

Benchmark of vertical excitation energies in the gas phase and implicit solvent with different levels of theory, structural parameters of equilibrium geometries computed on the S_0_ and S_1_ electronic states, comparison of structural parameters of conformational isomers isolated in the ground state, normalized distributions of selected structural parameters extracted from AIMD simulations for both molecular units, additional molecular orbitals involved in the first two CT electronic transitions and electronic density variation related to the CT (S_1_) state, normal mode compositions of the TCNE:π:1ClN complex computed in the ground state, and structural analysis of TCNE:M^+^ ground-state equilibrium geometries in acetonitrile solution (PDF)
