# Supplementary material for: Electronic and Vibrational Manifold of Tetracyanoethylene–Chloronaphthalene Charge Transfer Complex in Solution: Insights from TD-DFT and Ab Initio Molecular Dynamics
Source: J Phys Chem A. 2022 Sep 29;126(40):7179–92. doi: 10.1021/acs.jpca.2c05001 (PMC9574931; doi:10.1021/acs.jpca.2c05001)
Supplement: Supplementary file 1 — jp2c05001_si_001.pdf [file jp2c05001_si_001.pdf]

Supporting Information - Electronic and Vibrational Manifold of Tetracyanoethylene-Chloronaphthalene Charge Transfer Complex in Solution: Insights from TD-DFT and Ab Initio Molecular Dynamics

Federico Coppola,[†,‡] Paola Cimino,[¶] Fulvio Perrella,[†,‡] Luigi Crisci,[†] Alessio Petrone,[*,†,‡,§] and Nadia Rega[*,†,‖,‡,§]

†*Department of Chemical Sciences University of Napoli Federico II, Complesso Universitario di M.S. Angelo, via Cintia, Napoli 80126, Italy*

‡*Scuola Superiore Meridionale, Largo San Marcellino 10, I-80138, Napoli, Italy*

¶*Department of Pharmaceutical Sciences, University of Salerno, 84084,Fisciano (SA), Italy*

§*Istituto Nazionale Di Fisica Nucleare, sezione di Napoli, Complesso Universitario di Monte S. Angelo ed. 6, via Cintia, 80126, Napoli, Italia*

‖*CRIB, Centro Interdipartimentale di Ricerca sui Biomateriali, Piazzale Tecchio, I-80125, Napoli, Italy*

E-mail: *alessio.petrone@unina.it; *nadia.rega@unina.it

Contents

Table S1: Comparison of vertical excitation energies computed in gas and implicit solvent at different theory levels.

Table S2: Structural parameters of the TCNE:π:1ClN CT complex (Min_a) discussed in the main text.

Table S3: Main structural parameters and NBO population analysis calculated in implicit DCM solvent for three CT complexes ($Min_{a,b,c}$).

Figure S1: Orientational isomers of the TCNE:π:1ClN CT complexes ($Min_{a,b,c}$) computed in the ground and first excited states.

Figure S2: Bond lengths of TCNE and 1ClN monomers in the $Min_{a,b,c}$ CT complexes computed in the ground and first excited states.

Figure S3: Normalized distributions of selected structural parameters of TCNE monomer extracted from 10 ps AIMD trajectory sampled in the ground state.

Figure S4: Normalized distributions of selected structural parameters of 1ClN monomer extracted from 10 ps AIMD trajectory sampled in the ground state.

Figure S5: Contour plot of molecular frontier orbitals involved in the first two electronic transitions of the TCNE:π:1ClN CT complex.

Figure S6: Electronic density variation related to the first singlet excited state computed for the TCNE:π:1ClN CT complexes ($Min_{a,b,c}$).

Figure S7: Normal modes composition of TCNE:π:1ClN CT complex computed for the ground state minimum energy structure.

Figure S8: Structural parameters computed for the TCNE:M^+ geometries in ground state at UB3LYP/6-31+g(d,p)/C-PCM(ACN) potential.

Table S1. Vertical transition energies, in eV and nm, of the first five singlet excited states evaluated in gas phase and in implicit dichloromethane solvent with two different basis set. Oscillator strength f are given as pure number. Ground state minimum energy structures were computed at the corresponding theory level using the hybrid B3LYP functional. Experimental absorption values ($S_1 \leftarrow S_0$ 530 nm and $S_2 \leftarrow S_0$ 408 nm) are retrieved from Ref. 1.

	**CAM-B3LYP/6-31G(d,p)/GD3**			**CAM-B3LYP/6-31G(d,p)/DCM(C-PCM)/GD3**			**Δ(eV)**
	eV	nm	f	eV	nm	f	
$\mathbf{S_1}$	2.35	528.17	0.0979	2.28	543.64	0.1259	-0.07
$\mathbf{S_2}$	3.08	402.34	0.0001	3.02	410.10	0.0002	-0.06
$\mathbf{S_3}$	3.94	314.76	0.0112	3.92	316.01	0.0181	-0.02
$\mathbf{S_4}$	4.41	280.86	0.0512	4.35	285.07	0.1274	-0.06
$\mathbf{S_5}$	4.61	268.66	0.1219	4.56	272.03	0.3372	-0.05
	**CAM-B3LYP/6-31+G(d,p)/GD3**			**CAM-B3LYP/6-31+G(d,p)/DCM(C-PCM)/GD3**			
	eV	nm	f	eV	nm	f	
$\mathbf{S_1}$	2.27	545.89	0.0914	2.20	564.85	0.1252	-0.07
$\mathbf{S_2}$	3.02	410.29	0.0001	2.95	420.19	0.0004	-0.07
$\mathbf{S_3}$	3.87	320.40	0.0105	3.84	322.42	0.0179	-0.03
$\mathbf{S_4}$	4.33	286.01	0.0334	4.27	290.20	0.1064	-0.06
$\mathbf{S_5}$	4.53	273.59	0.2221	4.46	278.04	0.3905	-0.07

Table S2. Comparison of main structural parameters calculated in implicit DCM solvent for TCNE:π:1ClN CT complex (Min$_{S0,1a}$) computed in the ground and first singlet excited state at B3LYP/6-31+G(d,p)/GD3/C-PCM(DCM) and TD-CAM-B3LYP/6-31+G(d,p)/GD3/C-PCM(DCM) theory level, respectively. Bond lengths are given in Å, dihedral angles in degrees. For labeling please refer to Figure 1 in the main text.

	**Ground State (S_0)**	**Excited State (S_1)**
**TCNE**		
C_2=C_2'	1.378	1.429
C_1-C_2|C_1'-C_2'	1.427 | 1.427	1.410 | 1.409
C_2-C_3|C_2'-C_3'	1.430 | 1.429	1.408 | 1.409
C_1-N_1|C_1'-N_3	1.163 | 1.163	1.163 | 1.163
C_3-N_2|C_3'-N_4	1.163 | 1.163	1.164 | 1.163
N_1-C_2-N_2	116.74	118.18
N_3-C_2'-N_4	117.18	117.73
N_1C_2=$C_2'N_4$	176.11	175.66
N_3C_2=$C_2'N_2$	176.05	172.71
**1ClN**		
C_2-Cl	1.759	1.710
C_1-C_2	1.428	1.421
C_2-C_3	1.384	1.405
C_3-C_4	1.410	1.380
C_4-C_5	1.383	1.401
C_5-C_6	1.421	1.405
C_6-C_1	1.438	1.428
C_6-C_7	1.421	1.410
C_7-C_8	1.380	1.393
C_8-C_9	1.414	1.389
C_9-C_{10}	1.381	1.398
C_{10}-C_1	1.420	1.404
C_2-C_1-C_6-C_7	178.84	177.88
C_{10}-C_1-C_6-C_5	179.04	178.12

Table S3. Comparison of main structural parameters and NBO total charges calculated in implicit DCM solvent for three CT complexes ($\mathbf{Min}_{a,b,c}$) complex computed in the ground and first singlet excited state at B3LYP/6-31+G(d,p)/GD3/C-PCM(DCM) and TD-CAM-B3LYP/6-31+G(d,p)/GD3/C-PCM(DCM) theory level, respectively.

	Min_a	Min_b	Min_c
**Ground State (S_0)**			
Relative Energy (Kcal/mol)	//	0.94	0.43
C=C $_{(TCNE)}$ (Å)	1.378	1.377	1.376
C=C Bond Order	1.75	1.75	1.75
C-Cl $_{(1ClN)}$ (Å)	1.759	1.762	1.762
Center of Mass $_{(TCNE:1ClN)}$ (Å)	3.534	3.801	3.576
NBO total charge $_{(TCNE)}$	-0.083	-0.066	-0.073
NBO total charge $_{(1ClN)}$	0.083	0.066	0.073
**Excited State (S_1)**			
Relative Energy (Kcal/mol)	0.01	0.05	//
C=C $_{(TCNE)}$ (Å)	1.429	1.430	1.429
C=C Bond Order	1.53	1.52	1.52
C-Cl $_{(1ClN)}$ (Å)	1.710	1.710	1.710
Center of Mass $_{(TCNE:1ClN)}$ (Å)	3.469	3.344	3.469
NBO total charge $_{(TCNE)}$	-0.840	-0.854	-0.870
NBO total charge $_{(1ClN)}$	0.840	0.854	0.870
$\mathrm{d}^{CT}{}_{(TCNE:1ClN)}$ (Å)	2.506	2.489	2.580
$\mathrm{q}^{CT}{}_{(TCNE:1ClN)}$	0.968	1.009	1.026

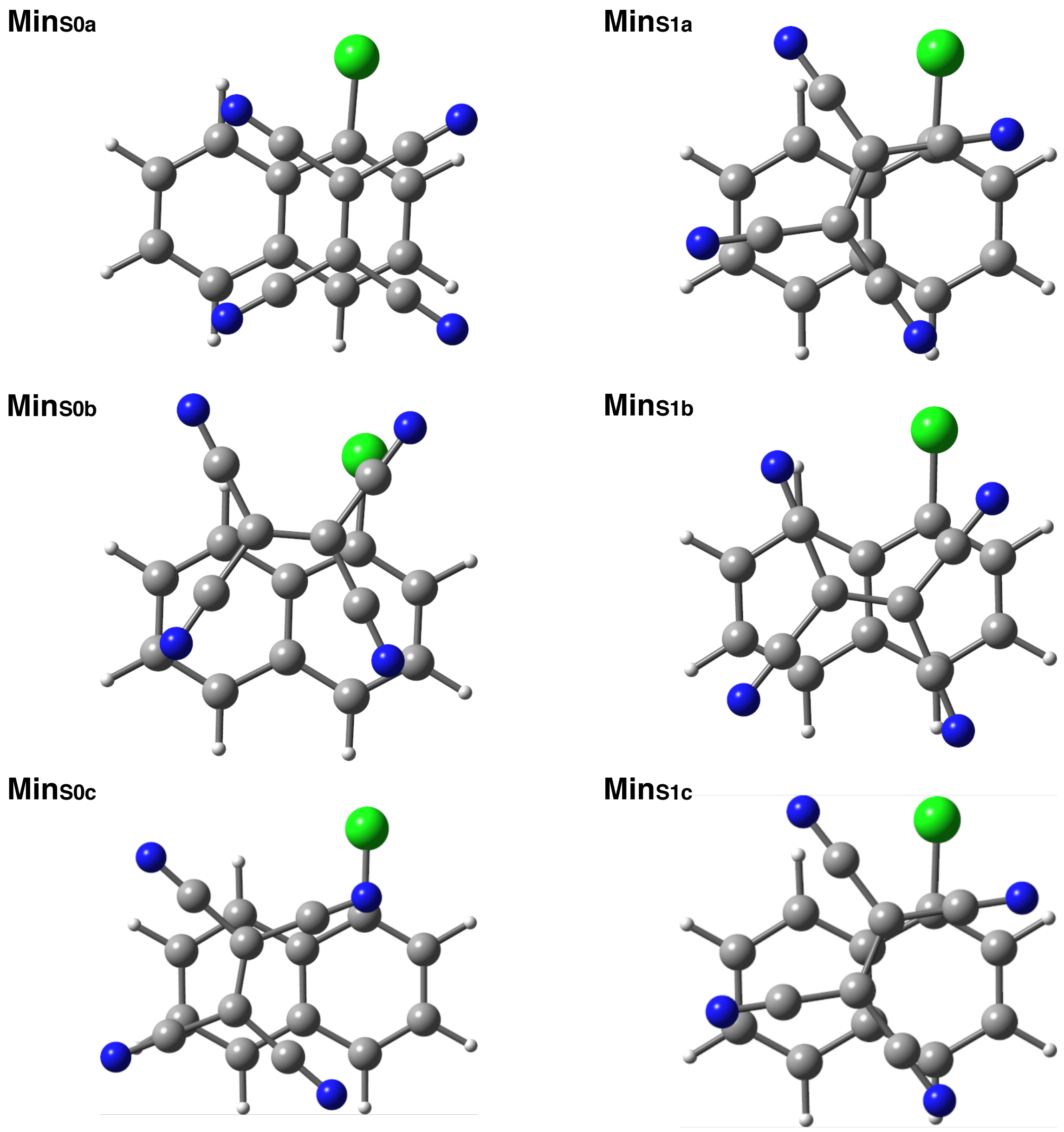

Figure S1. Top view of the TCNE:π:1ClN charge transfer dimers computed in S_0 and S_1 electronic states.

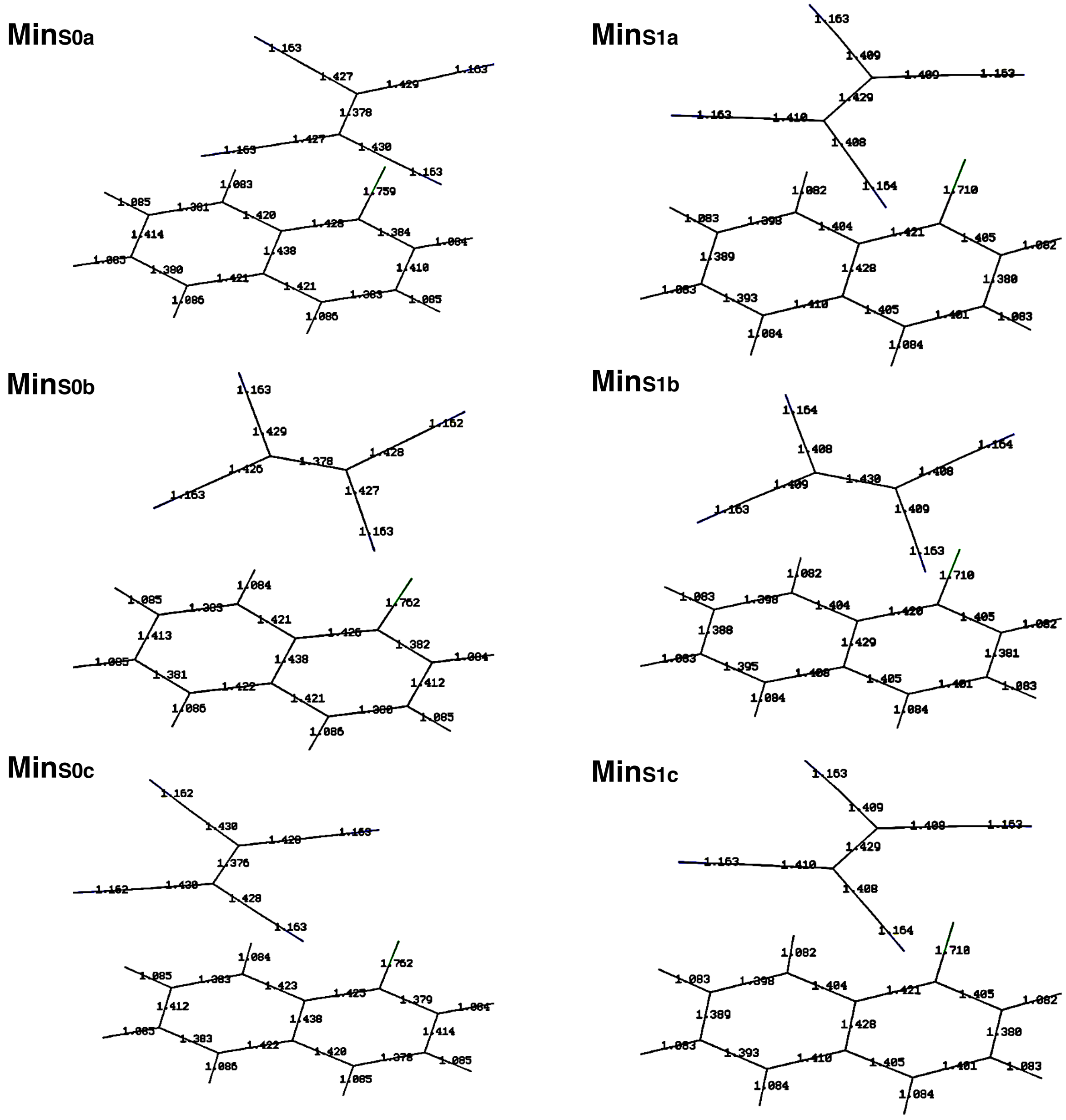

Figure S2. Bond lengths of the TCNE:π:1ClN optimized structures in implicit dichloromethane solution in the ground (left) and first singlet excited state (right).

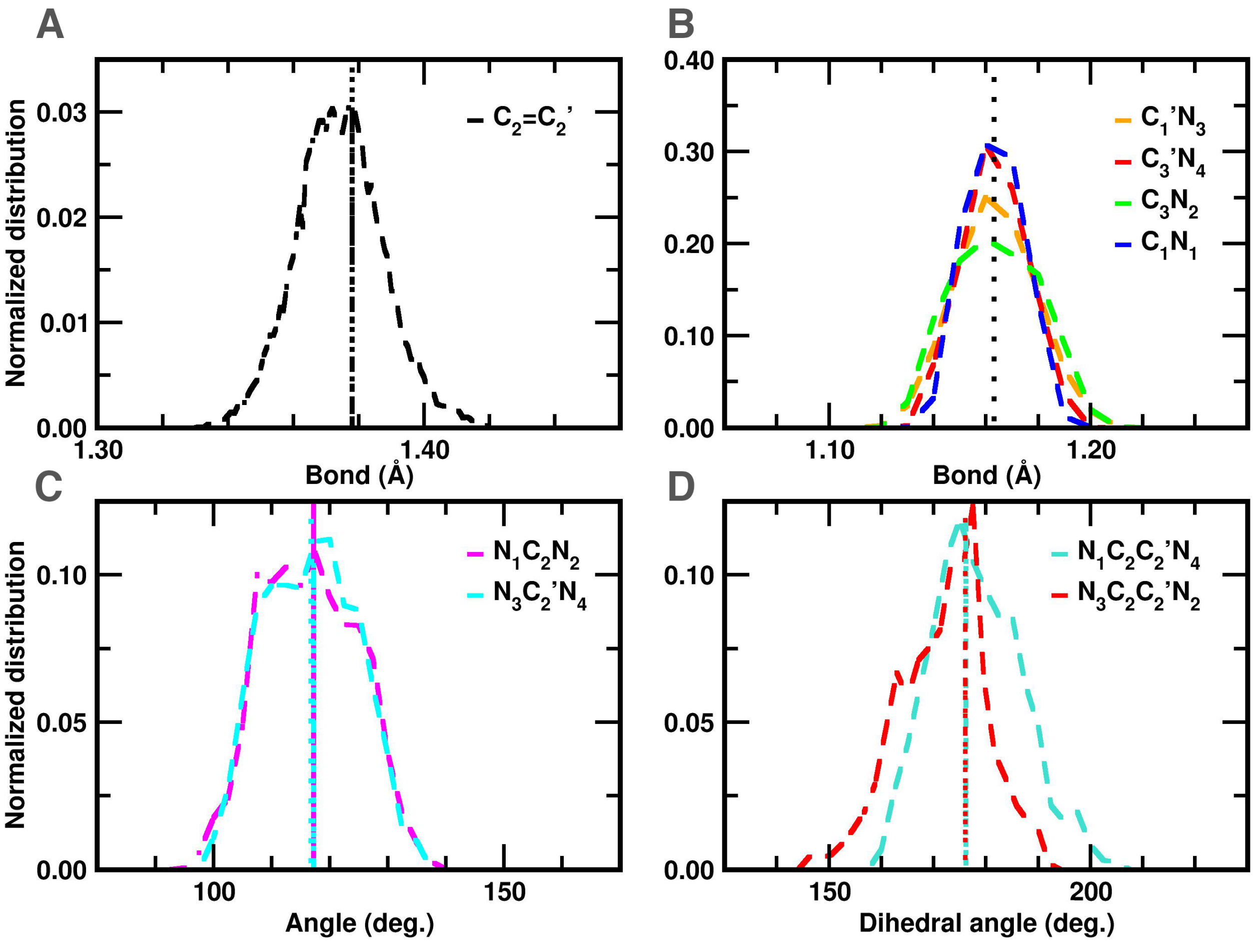

Figure S3. Normalized distributions at 0.001 Å resolution for bond distances, and 2.5° for angles and dihedrals of selected structural parameters of TCNE acceptor monomer extracted from 10-ps long AIMD trajectory sampled in the ground state. Panel **A**, C=C central double bond length; panel **B**, C≡N bond lengths; panel **C**, N-C-N angles; panel **D**, N-C-C-N dihedral angles. Vertical dashed line refers to structural parameters of Min_{S0a} for comparison. For labeling scheme refer to Figure 1 in the main text.

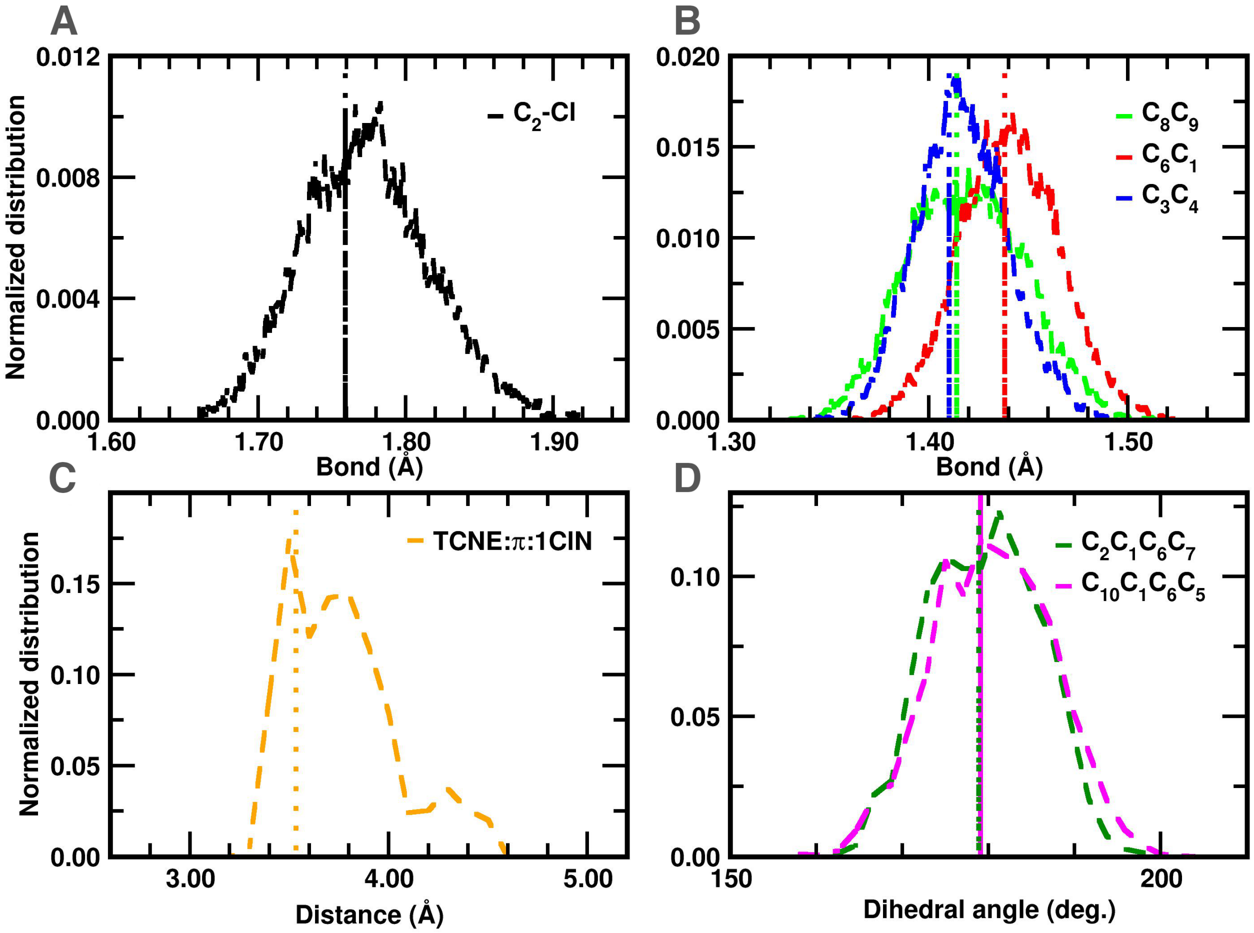

Figure S4. Normalized distributions at 0.001 Å resolution for bond distances, and 2.5° for angles and dihedrals of selected structural parameters of 1ClN donor monomer extracted from 10-ps long AIMD trajectory sampled in the ground state. Panel **A**, C-Cl bond length; panel **B**, ring central C-C bond lengths; panel **C**, TCNE:π:1ClN center of mass distance (0.02 Å resolution); panel **D**, C-C-C-C dihedral angles. Vertical dashed line refers to structural parameters of Min_{S0a} for comparison. For labeling scheme refer to Figure 1 in the main text.

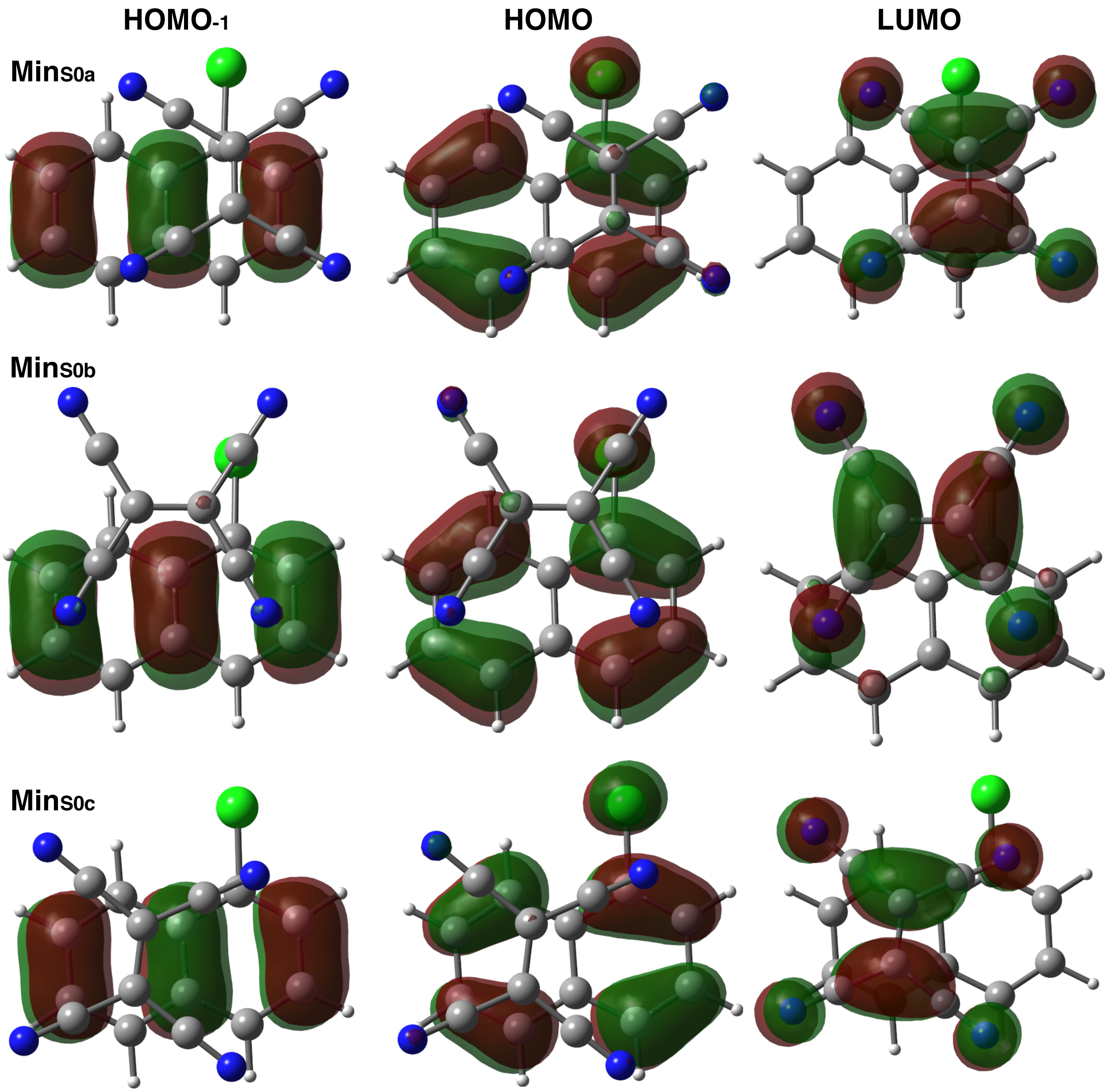

Figure S5. Molecular frontier orbitals counturs (isovalue 0.03) involved in $S_1 \leftarrow S_0$ (HOMO-LUMO) and $S_2 \leftarrow S_0$ ($HOMO_1$-LUMO) vertical excitation for the three ground state conformers investigated, $\mathrm{Min}_{S0a,b,c}$.

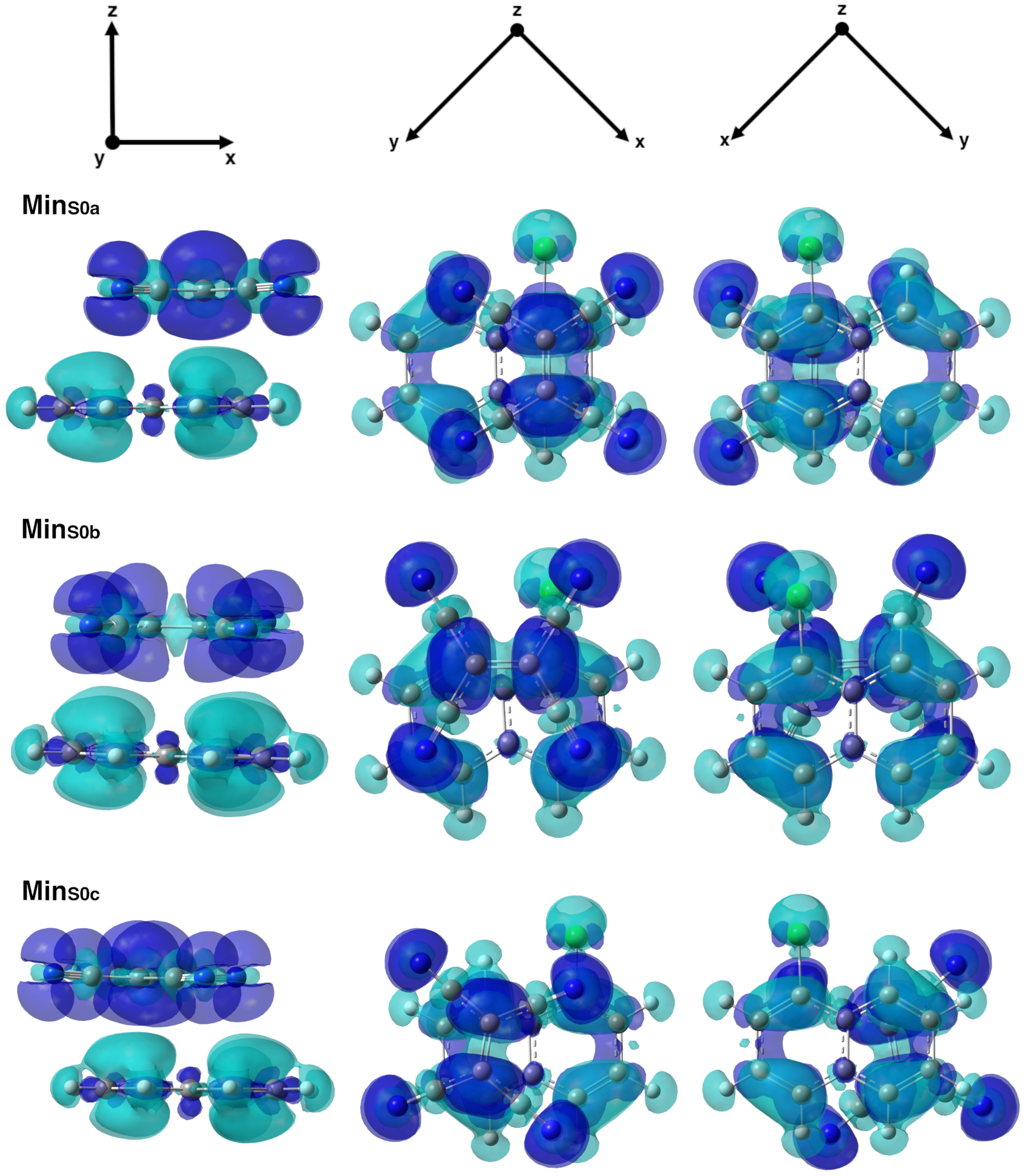

Figure S6. Positive and negative variation of electronic density in dark and light blue, respectively, associated to the first vertical excitation of the TCNE:π:1ClN charge transfer dimer.

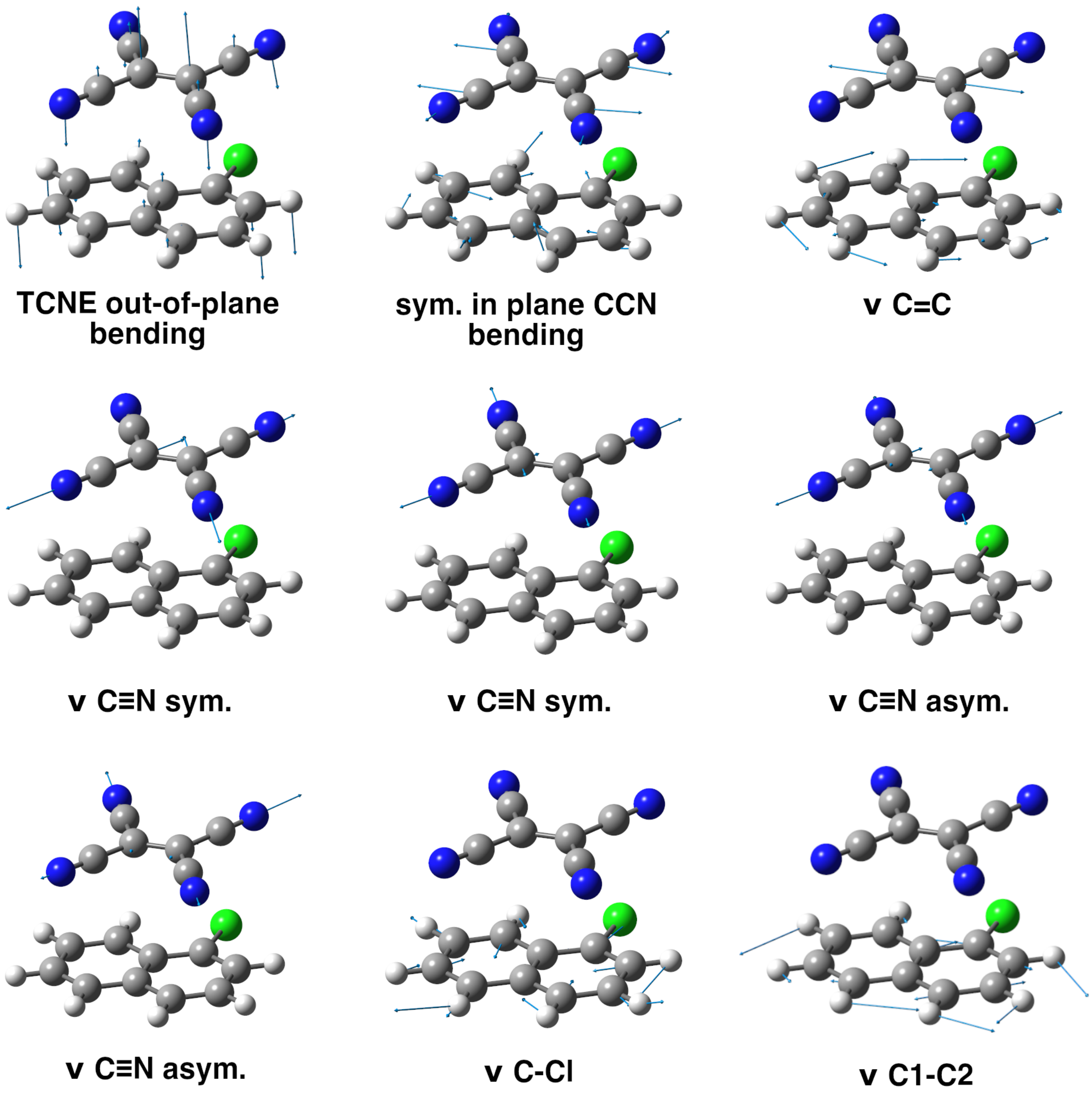

Figure S7. TCNE:π:1ClN CT complex normal mode displacement vectors computed in ground state at B3LYP/6-31+G(d,p)/DCM(C-PCM)/GD3 potential. Harmonic and anharmonic vibrational frequencies are reported in Table 3 in the main text.

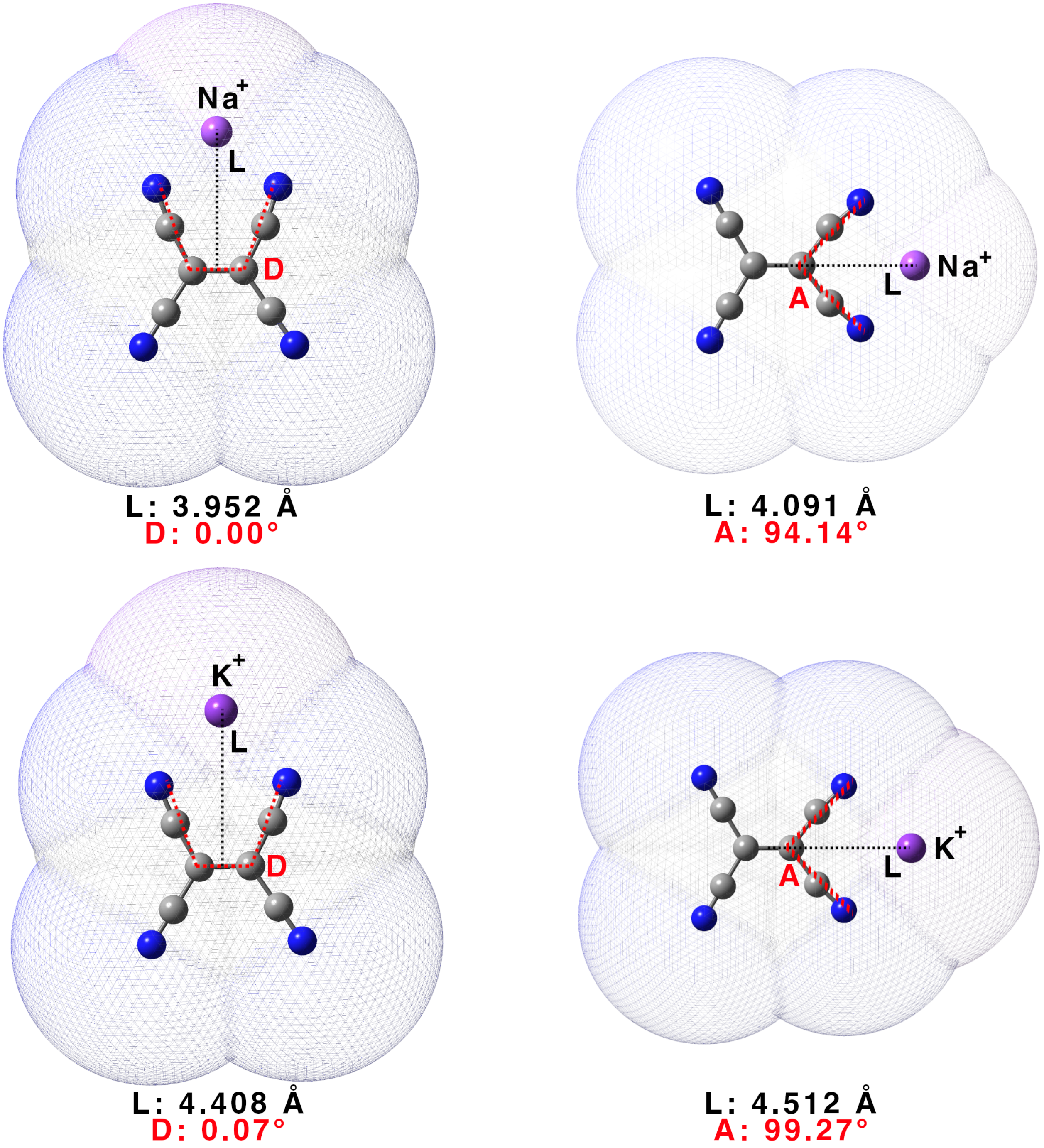

Figure S8. Structural parameters computed for the TCNE:M^+ geometries in ground state at UB3LYP/6-31+g(d,p)/C-PCM(ACN) potential. The C-PCM acetonitrile Solvent Accessible Surface cavity is also shown. **L** is the distance from the central C=C bond (in Å), **D** is the NC=CN dihedral angle facing the counterion and analogously **A** is the NCN angle (in deg.). Left: the counterion is in the C-C=C-C *major* groove, right: the counterion is in the C-C-C *minor* groove.

References

(1) Ellis, S. R.; Hoffman, D. P.; Park, M.; Mathies, R. A. Difference Bands in Time-Resolved Femtosecond Stimulated Raman Spectra of Photoexcited Intermolecular Electron Transfer from Chloronaphthalene to Tetracyanoethylene. *J. Phys. Chem. A* **2018**, *122*, 3594–3605.
